# Prevalence, time trends and associated factors of adult overweight and obesity in 36 countries in the WHO African region from 2003 to 2022: a study of 54 WHO STEPS surveys representing 156 million adults

**DOI:** 10.1136/bmjgh-2025-019988

**Published:** 2026-01-13

**Authors:** Kouamivi Mawuenyegan Agboyibor, Aboubakari Nambiema, Ali Golestani, Joseph Okeibunor, Cheick Bady Bady Diallo, Xavier Jouven, Jean-Marie Dangou, Farshad Farzadfar, Jean-Philippe Empana, African STEPS Group, Leanne Riley

**Affiliations:** 1 Inserm, PARCC, F-75015, Université Paris Cité, Paris, Île-de-France, France; 2 Noncommunicable disease, World Health Organization, Geneva, GE, Switzerland; 3 PARCC, INSERM, Paris, Île-de-France, France; 4 Tehran University of Medical Sciences, Tehran, Iran (the Islamic Republic of); 5 Emergency Preparedness and Response, World Health Organization Regional Office for Africa, Brazzaville, Brazzaville, Congo; 6 NMH, PAHO, Washington, District of Columbia, USA; 7 Noncommunicable disease, World Health Organization Regional Office for Africa, Brazzaville, Brazzaville, Republic of Congo

**Keywords:** Cross-sectional survey, Africa South of the Sahara, Nutritional and metabolic disorders, Public Health, Epidemiology

## Abstract

**Background:**

We investigated the prevalence, temporal trends and associated factors of overweight and obesity among adults in the WHO African region.

**Methods:**

We analysed individual-level data from 54 nationally/sub-nationally representative STEPS surveys conducted between 2003 and 2022 among adults aged 18–69 years. Prevalence estimates were weighted and age-standardised. Time trends were estimated using a Bayesian spatiotemporal modelling approach. Factors associated with body mass index (BMI) categories were identified in hierarchical multinomial mixed-effects logistic regression with random effects for country and survey year, using the normo-weighted as the reference group.

**Results:**

The study population included 198 901 adults (50.3% women) with a mean age of 36.3 years. The mean BMI was 23.3±2.0 kg/m^2^ (24.23±1.60 in women and 22.11±1.53 in men, p for sex difference <0.001). The prevalence of overweight and obesity was 17.8% and 9.0%, respectively, higher in women (20.8% and 13.3%) than in men (14.9% and 4.6%). There was no significant time trend in mean BMI (23.25 kg/m² (95% CI 20.1 to 26.6) in 2003 and 23.43 kg/m² (95% CI 19.3 to 27.8) in 2022, p for trend=0.75). However, obesity prevalence increased from 15.39% to 16.71% (p for trend <0.001), and underweight from 12.07% to 12.76% (p for trend <0.001), whereas overweight plateaued. In multivariate analysis, sex, older age, higher education, physical inactivity and low fruit and vegetable consumption increased the odds of overweight and obesity, whereas past and current smoking showed inverse associations. Specifically, adjusted odds ratios for overweight and obesity for females versus males were 2·07 [(95% CI: 1·83– to 2·34]) and 4.92 [(95% CI: 4·13– to 5·89]); for tertiary education versus no education, they were 2·07 [(95% CI: 1·63– to 2·63]) and 3·77 [(95% CI: 2·77– to 5·11]), respectively.

**Conclusion:**

These findings support the urgent need to intensify preventive programmes to fight obesity in the WHO African region.

**WHAT IS ALREADY KNOWN ON THIS TOPIC**

- The increase in obesity prevalence has been recognised as one of the major contributing factors to the significant rise in many non-communicable diseases (NCDs) globally, including in Africa. Addressing obesity is crucial to achieving the United Nations Sustainable Development Goal of a 33% reduction in premature mortality from NCDs by 2030 and the WHO’s target of halting the rise in obesity by 2025.
- Several large-scale studies by the Non-Communicable Disease Risk Factor Collaboration (NCD-RisC) have examined global trends in body mass index (BMI) and the burden of overweight and obesity, including in Africa. However, while the use of multiple data sources permits getting closer to exhaustivity, this can be offset by the heterogeneity and unharmonised data sources.
- Six multi-country studies using Demographic Health Survey (DHS) data have assessed overweight and obesity in African women, primarily in urban areas, while two systematic reviews have focused on specific subpopulations with high BMI or type 2 diabetes. These prior studies do not adequately represent the burden of obesity in the general African adult population.

**WHAT THIS STUDY ADDS**

**HOW THIS STUDY MIGHT AFFECT RESEARCH, PRACTICE OR POLICY**

- This study underscores the significant and growing burden of obesity among adults in the WHO African region. In particular, it highlights that females are disproportionately affected compared to males, emphasising the need for sex- and gender-specific policies and interventions.
- The findings support urgent public health action, including tailored prevention strategies at both national and regional levels, to address this escalating challenge and contribute towards achieving global NCD targets.

## Introduction

 Overweight and obesity, defined as body mass index (BMI) between 25 to <30 kg/m^2^ (overweight), and ≥30 kg/m^2^ (obesity), are significant contributing factors for various chronic diseases such as cardiovascular diseases, type 2 diabetes, musculoskeletal disorders and some cancers. Overweight, obesity and its related conditions lead to reduced quality of life and premature death.^1^

The global prevalence of overweight and obesity has more than doubled over the past four decades. In 2022, 2.5 billion adults (18 years and older) were overweight, representing 43% of the global adult population, with 890 million of them living with obesity.^2 3^ Recent studies indicate that while the rate of increase in overweight and obesity may be slowing in high-income countries, the epidemic appears to be accelerating in low- and middle-income countries (LMICs), where about 67% of individuals with obesity now live.^4^,^8^

In Africa, projections suggest a notable rise in obesity among children and women by 2035, leading to significant economic implications.^9^ For women, the prevalence of obesity is anticipated to rise from 18% in 2020 to 31% by 2035. The annual economic impact is likely to reach over US$50 billion per year by 2035, or 1.6% of the region’s gross domestic product (GDP).^10^ The WHO African region, which comprises 47 countries primarily in sub-Saharan Africa, with Algeria as the only North African country included**,**^11^ faces multifaceted challenges in combatting overweight/obesity, including fragile healthcare systems, limited resources, socioeconomic complexities, urbanisation and insufficient public health policies.^112^,^14^

Despite growing concerns, comprehensive studies on obesity across multiple African countries, with consistent data sources and methodologies, remain limited. The Non-Communicable Disease Risk Factors Collaboration (NCD-RisC) has regularly and extensively updated the burden of obesity and overweight worldwide, including in the WHO African region.^6^,^8^ However, and despite rigorous statistical methodology, the analysis combined data from multiple sources and study design with heterogeneous data collection methodologies. Furthermore, the previous largest multi-country study on the burden of overweight and obesity in Africa comprised 24 countries and focused on women only from urban areas.^15^ Two prior systematic reviews in Africa addressed the burden of obesity but in clinical samples.^16 17^ Finally, the place of contextual factors as potential actionable determinants of obesity in Africa is understudied.

Therefore, the main objective of this study was to provide an extensive estimation of the burden of overweight and obesity in 36 countries from the WHO African region. Secondary objectives were to describe trends in mean BMI and BMI categories over 20 years and to identify both individual and contextual factors associated with overweight and obesity in the WHO African region.

## Methods

### Study design and data source

Among the 47 countries in the WHO African region, 38 had conducted at least one WHO STEPS (STEPwise approach to NCD risk factor surveillance) survey, while nine had not conducted the survey as of the time of our request. We obtained access to surveys from 37 countries available in the WHO STEPwise survey repository, though one had missing BMI data. We analysed individual-level data from the remaining 36 countries, combining 54 subnational and national cross-sectional WHO STEPwise surveys conducted between 2003 and 2022. The list and description of available STEPS surveys by country is reported in online supplemental table 1. The STEPS is a standardised tool developed by WHO and partners to gather information on the prevalence of non-communicable disease (NCD) risk factors in countries. The design of the STEPS has been published elsewhere.^18^ Briefly, the survey is conducted in three steps: step 1, a questionnaire-based interview to collect information on demographic characteristics and risk factors for NCDs; step 2, physical measurements such as height, weight and blood pressure; and step 3, biochemical measurements such as blood glucose levels, blood lipid levels and urinary sodium levels (online supplemental appendix 1).

### Definitions

- Body mass index (BMI):

BMI is a widely used metric to classify underweight, normal weight, overweight and obesity in adults. It is calculated by dividing a person’s weight in kilograms by the square of their height in metres (kg/m²). According to WHO, BMI categories are defined as follows:

- Underweight: BMI <18.5
- Normal weight: BMI 18.5–24.9
- Overweight: BMI 25–29.9
- Obesity: BMI ≥30.

- Physical activity:

Physical activity was evaluated by questionnaire and three levels of activity were defined:

- *Low physical activity*: 0 min of activity, or 1–149 min per week of moderate-intensity activity, or 1–74 min per week of vigorous-intensity activity, or a combination of 1–149 min per week of both.
- *Moderate physical activity*: 150 min or more per week of moderate-intensity activity, or 75 min or more per week of vigorous-intensity activity, or a combination of ≥150 min of both moderate and vigorous activity.
- *High physical activity*: Engaging in more than the minimum recommendations (eg, ≥300 min per week of moderate-intensity or ≥150 min per week of vigorous-intensity activity).

- Diet:

We have defined diet according to the daily portions of fruits and vegetables intake. Three levels were considered:

- *Low diet*: 0–1 portions daily
- *Moderate diet*: 2–3 portions daily
- *High diet*: 4–5 portions daily.

Additional covariate definitions are provided in the online supplemental appendix 2.

### Statistical analysis

#### Prevalence of overweight and obesity

Weighted prevalences of overweight and obesity accounted for the study sampling in each country. They were also age-standardised with the direct method using the African population as reference.^19^ Confidence intervals were estimated by the Taylor series linearisation method, which adjusts for the complex survey design to provide accurate standard errors.^20^

#### Trends in BMI

The methods used to estimate trends in BMI and BMI categories over the 20-year period (2003 to 2022) have been previously described and are detailed in online supplemental appendix 3.^21 22^ Importantly, this analysis was conducted using country level aggregated data including BMI together with data from the World Bank and the United Nations Development Programme (UNDP) (see below). In particular, this means that this method estimates BMI at the country level and not at the individual level. Since BMI data were assessed at one single time point for most countries, we imputed aggregated BMI data both at the spatial (ie, country level) and temporal (ie, over time) level using a spatiotemporal model. Notably, the spatiotemporal analysis enabled us to extend BMI prevalence estimates to the 11 African countries for which STEPS data were not available, thereby providing trends for all 47 member states in the WHO African region. This model incorporates covariates from the World Bank and UNDP, including the Food Production Index, GDP per capita, grams of fat per day per capita, prevalence of undernourishment, employment in agriculture, value added in agriculture and urban population to impute missing BMI data.^23 24^ The analysis involved three major steps. First, missing World Bank and UNDP covariate data were imputed using the Amelia multiple imputations package.^25^ Second, the most relevant World Bank and UNDP covariates for predicting BMI were identified using stepwise selection based on the Akaike Information Criterion. Finally, trends over the 20-year period (2003 to 2022) in mean aggregated BMI and in the prevalence of BMI categories were estimated using a spatiotemporal Bayesian model defined using INLA (Integrated Nested Laplace Approximation). To assess the statistical significance of the trends, we fitted separate unadjusted linear regression models with the mean aggregated BMI and the prevalence of each BMI category as the dependent variables, and year as the independent variable. We then extracted the p values associated with the regression coefficient for year.

#### Factors associated with BMI categories

Since we used normo-weighted individuals as the reference category, hierarchical (multilevel) multinomial mixed-effects logistic regression models were employed to explore factors associated with underweight, overweight and obese. Also, these group comparisons aligned with public health practice. The models accounted for individuals nested within countries, with random intercepts for both country and survey year. These random effects were assumed to be normally distributed, allowing for variability across countries and years in the associations between covariates and BMI categories.^26 27^ The models adjusted for individual-level covariates such as sex, age, education level, smoking, alcohol consumption, physical activity and fruit and vegetable consumption.

#### Exploratory analysis

To evaluate the possible contribution of contextual factors, we further adjusted the hierarchical multinomial mixed-effects logistic regression for contextual data such as the World Bank and UNDP data. These include unemployment rate, agricultural productivity, urbanisation percentage, GDP per capita, life expectancy, years of schooling, the Gender Development Index (GDI), and female participation in the workforce at the country level. Only those that were related to overweight and obesity in univariate analysis (p value <5%) were thereafter considered in the multivariable model.

In the subsample of surveys containing information on rural residency and including 111 577 participants, we depicted the distribution of BMI categories by urban/rural status and further adjusted the multivariable hierarchical (multilevel) multinomial mixed-effects model on the rural/urban status.

To assess the public health impact of our findings, we estimated the population attributable fraction (PAF) of obesity and overweight (exposure) for adverse blood pressure and type 2 diabetes (outcomes). We first quantified the association of BMI categories with adverse blood pressure and type 2 diabetes using multilevel mixed multivariate logistic regression adjusted for sex, age, education level, smoking, alcohol consumption, physical activity and fruit and vegetable consumption. Then, the weighted prevalence of overweight and obesity and the odds ratios (OR) estimated above were used to address the PAFs using the formula developed and popularised by Levin (1953), specifically in the context of epidemiological studies.^28 29^


$$PAF=\frac{p_{e}(OR-1)}{p_{e}(OR-1)+1}$$

All statistical analyses were performed using R software, version 2024.12.1+402.

## Results

We compiled data from 198 901 adult participants (50.3% women) with a mean age of 36.3±12.9 years, representing a population of 156 million from 54 surveys. Of the 36 countries examined, 13 were in West Africa, nine in East Africa, five in Southern Africa, eight in Central Africa and one in North Africa (Algeria only north African country member of the WHO African region). The study flow chart and a map of the countries covered in this study are respectively depicted in online supplemental figure 1 and 2).

### Baseline characteristics

**Figure 1:**
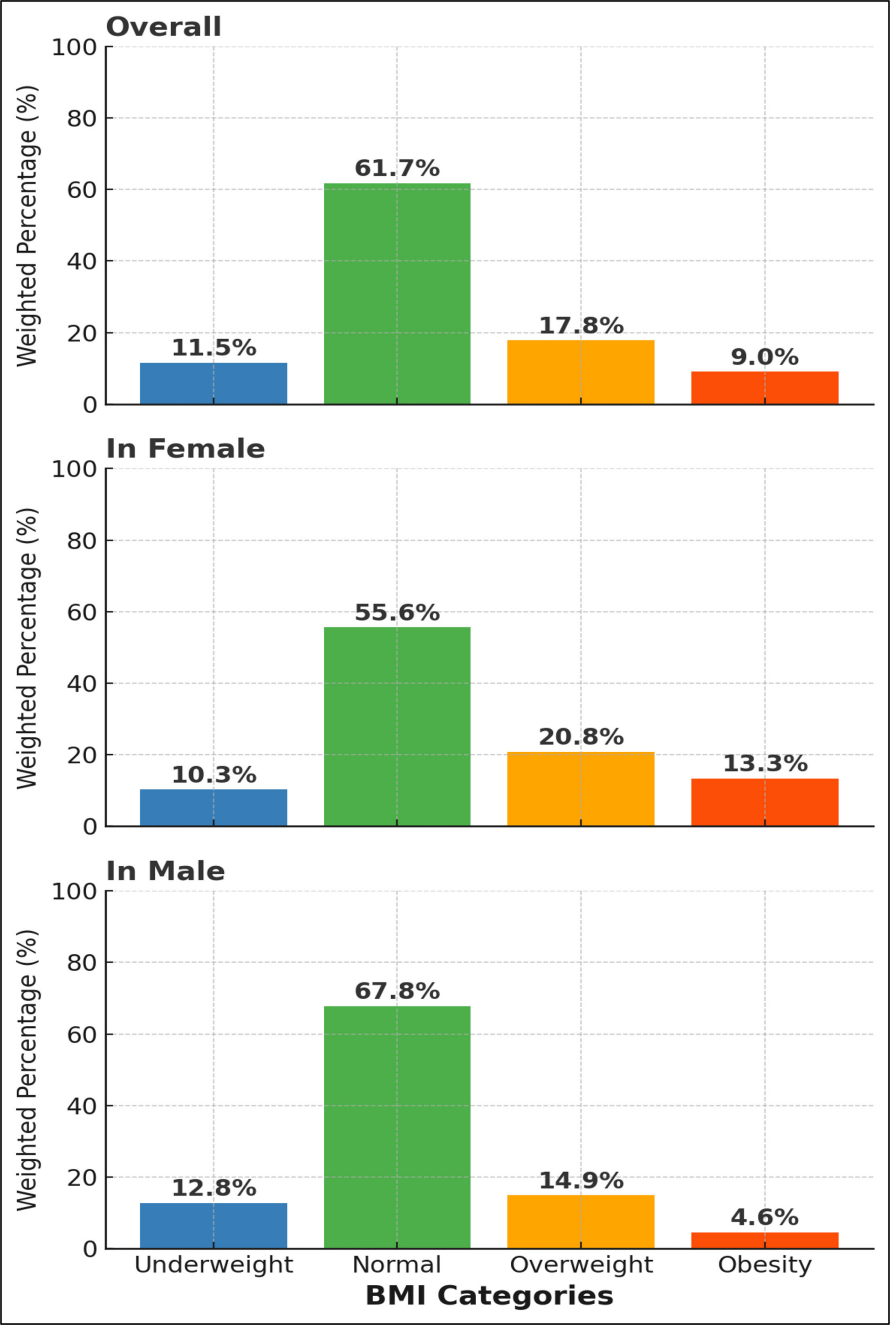
Distribution of prevalence of body mass index (BMI) categories overall and by sex. The total reaches 100% in each sex.

The overall mean (SD) BMI was 23.3±2.0 kg/m^2^, 24.23±1.60 in women and 22.11±1.53 in men (p for sex difference <0.001). The age-standardised and weighted prevalence of underweight, overweight and obesity was 11.5%, 17.8% and 9.0%, respectively, and 61.7% were normo-weighted. The corresponding unweighted prevalences are reported in online supplemental table 2. Women were three times more often obese and 1.4 times more often overweight than men (figure 1). The distribution of the BMI categories by study periods and by subregions is shown in online supplemental figures 3 and 4. The distributions of mean BMI (overall and by sex) and BMI categories (overall and by sex) by country and by year are reported in the supplementary material (online supplemental figures 8–16).

The distribution of individual characteristics overall and by BMI categories is reported in table 1 and online supplemental table 3. In addition to the previously noted sex disparities, we noticed that overweight and obese individuals, as compared with normo-weighted individuals, were older, more educated, less physically active, had less often optimal diet, were more often never or past smokers, alcohol abstainers and had higher levels of blood pressure, total cholesterol and blood glycaemia.

**Table 1:** Characteristics of study participants overall and by BMI categories

Characteristic		BMI (kg/m^2^)				
		Overall, n=198 901 (100%)*	Underweight (<18.5), n=20 991 (11.5%)†	Normal (18.5 to 24.9), n=114 560 (61.7%)†	Overweight (25 to 29.9), n=40 470 (17.8%)†	Obese (≥30), n=22 880 (9.0%)†
Female sex	198 901	115 811 (50.3%)	11 499 (10.3%)	59 543 (55.6%)	26 319 (20.8%)	18 450 (13.3%)
Age, mean (SD)	195 633	36.3 (12.9)	36.0 (13.9)	34.8 (12.6)	38.8 (12.5)	41.3 (12.0)
Level of education	193 950					
No education		61 605 (26.7%)	8581 (16.3%)	36 681 (63.5%)	10 657 (13.4%)	5686 (6.7%)
Primary education		74 133 (44.4%)	7301 (11.2%)	43 734 (63.8%)	14 586 (16.8%)	8512 (8.3%)
Secondary education		45 623 (22.2%)	3650 (8.3%)	24 969 (58.5%)	10 730 (22.0%)	6274 (11.2%)
Tertiary education		12 589 (6.6%)	900 (5.6%)	6369 (50.9%)	3332 (28.3%)	1988 (15.2%)
Physical activity	193 202					
≥150 min/week moderate or ≥75 min/week vigorous or ≥150 combination		75 255 (53.6%)	7912 (11.6%)	45 742 (64.3%)	14 332 (16.6%)	7269 (7.5%)
Fruit and vegetable consumption	165 516					
4–5 servings per day		87 275 (51.7%)	9784 (12.8%)	51 112 (63.1%)	17 026 (16.3%)	9353 (7.9%)
Smoking	192 868					
Never or quit >12 months		169 349 (87.4%)	17 046 (11.1%)	95 774 (60.8%)	35 817 (18.5%)	20 712 (9.6%)
Alcohol consumption	193 404					
Never or quit >12 months		113 391 (60.4%)	12 304 (10.8%)	63 704 (58.7%)	23 491 (19.9%)	13 892 (10.6%)
Systolic blood pressure, mm Hg	193 275	126.4 (31.0)	120.7 (30.7)	125.4 (32.8)	130.2 (25.7)	133.4 (25.8)
Fasting blood glucose, mg/dL	138 056	80.0 (32.4)	76.4 (29.6)	77.0 (30.2)	86.5 (34.2)	93.1 (40.4)
Blood total cholesterol, mg/dL	112 328	136.2 (54.4)	125.8 (48.7)	129.6 (51.2)	152.6 (56.8)	163.3 (61.9)

All percentages are weighted; all n are unweighted.

* Mean (SD).

† Percentage in column (%); percentage in row.

BMI, body mass index.

#### Trends analysis

**Figure 2:**
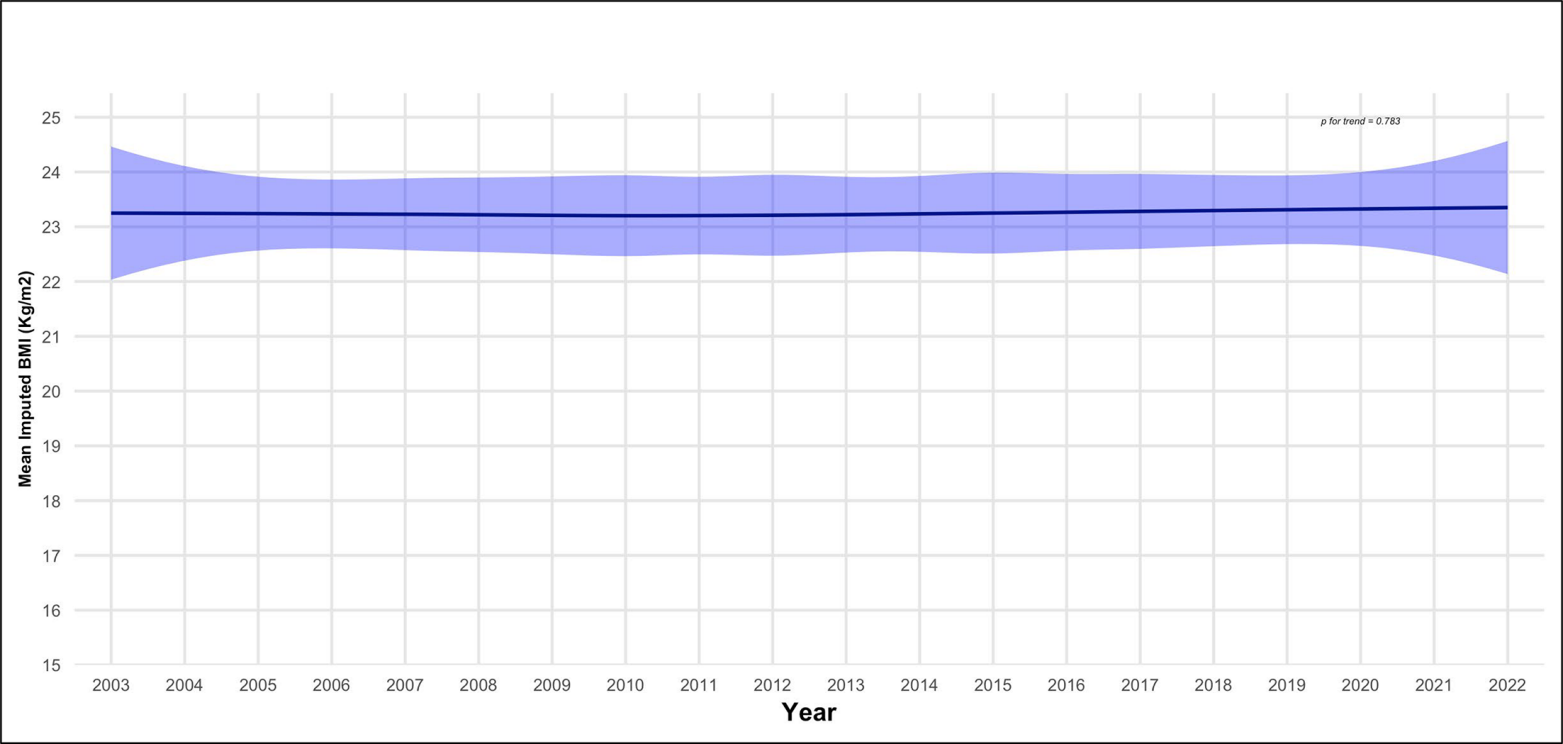
Trend in overall age-standardised mean body mass index (BMI) from 2003 to 2022. Imputed data for all the 47 countries in the WHO African region.

**Figure 3:**
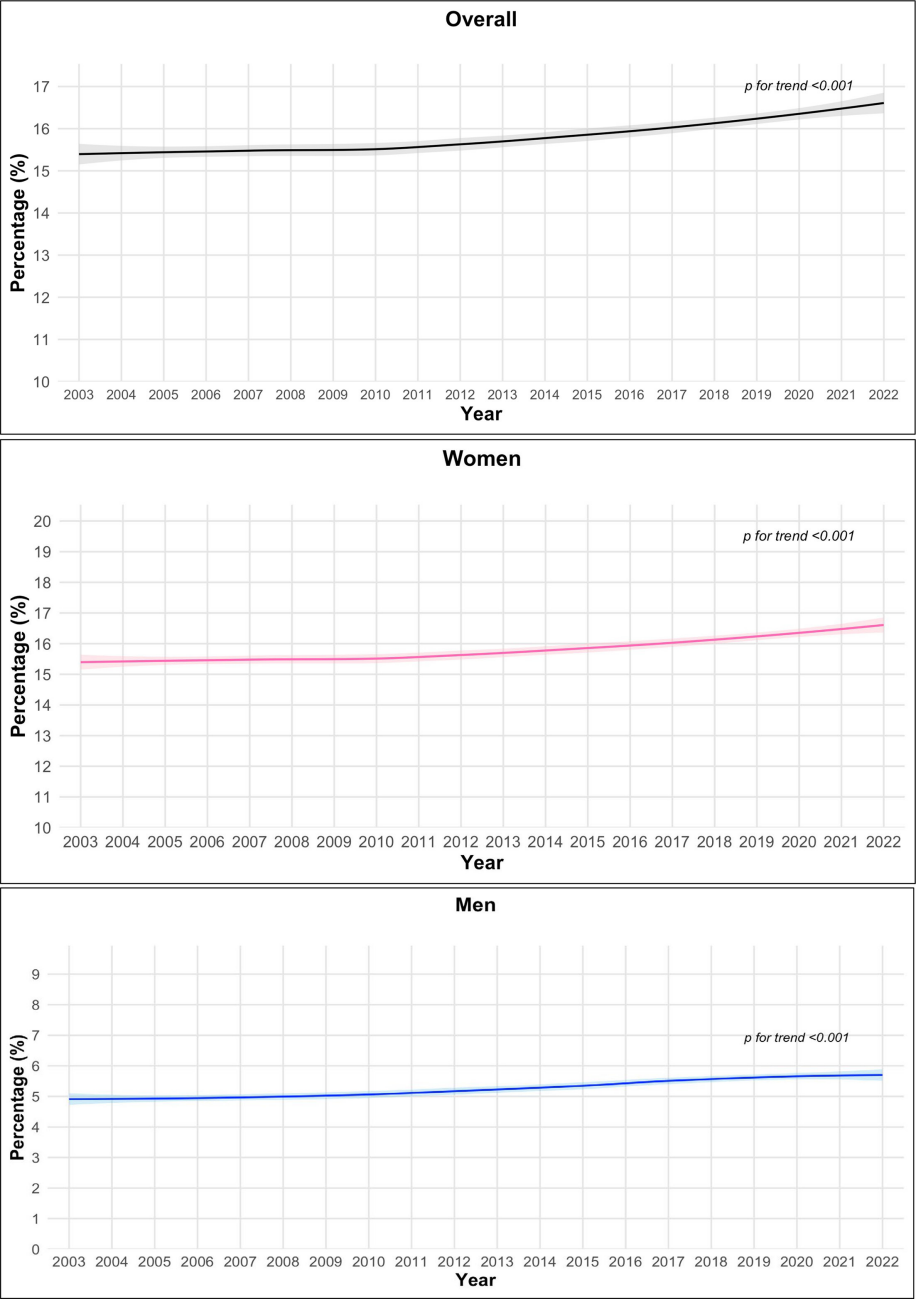
Trend in age-standardised prevalence of obesity overall and by sex from 2003 to 2022. Imputed data for all the 47 countries in the WHO African region.

Using the spatio-temporal model, no significant trend in overall mean BMI was noticed (figure 2), weighted and age standardised mean BMI being 23.25 kg/m^2^ (95% CI 20.1 to 26.6) in 2003 and 23.43 kg/m^2^ (95% CI 19.3 to 27.8) in 2022 (p for trend=0.783). This reflects a significant increase in the overall prevalence of obesity from 15.39% in 2003 to 16.71% in 2022 (p for trend<0.001) (figure 3) and underweight from 12.07% in 2003 to 12.76% in 2022 (p for trend <0.001) (online supplemental figure 7), along with a plateau in the prevalence of overweight from 18.01% in 2003 to 18.03% in 2022 (p for trend=0.461) (online supplemental figure 6).

Trend analysis by sex indicates that the prevalence of obesity increased from 15·31% in 2003 to 16·77% in 2022 in women (p for trend <0·001), and from 05·02% in 2003 to 05·90% in 2022 in men (p for trend <0·001) (figure 3). The corresponding sex-specific trends for overweight and underweight are reported in online supplemental figures 6 and 7, while the sex-specific trends for mean BMI are shown in online supplemental figures 17 and 18, respectively.

#### Risk factors for BMI categories

Factors associated with BMI categories are reported in table 2. After multivariable analysis, being a female, older age, higher education, physical inactivity and poor diet were positively associated with overweight or obesity compared with normal weight, whereas inverse associations were seen with past or current smoking. Strikingly, the odds of being overweight or obese were respectively two times higher (adjusted OR (aOR) 2·07, 95% CI: 1·83– to 2·34) and almost five times higher (aOR 4.92, 95% CI: 4·13– to 5·89) in female compared to males. Additionally, those with tertiary education were two times (aOR 2·07, 95% CI 1·63– to 2·63) and three times (aOR 3·77, 95% CI 2·77– to 5·11) more likely to be overweight and obese than less educated. Furthermore, older age and former and current smoking were associated with higher odds of underweight, whereas higher education was inversely related to underweight. Factors associated with BMI categories did not differ by sex (online supplemental table 4).FO

**Table 2:** Factors associated with overweight and obese status as compared with normal weight

Predictor	Underweight vs normal		Overweight vs normal		Obesity vs normal	
	OR	95% CI	OR	95% CI	OR	95% CI
	0.09	0.05 to 0.16	0.1	0.06 to 0.15	0.01	0.01 to 0.02
Sex						
Male	1 (ref)		1 (ref)		1 (ref)	
Female	1.25	1.06 to 1.46	2.07	1.83 to 2.34	4.92	4.13 to 5.89
Age groups (years)						
18 to 25	1 (ref)		1 (ref)		1 (ref)	
25 to 35	0.85	0.67 to 1.08	1.85	1.49 to 2.29	2.83	2 to 4.06
35 to 45	0.88	0.68 to 1.14	2.44	1.96 to 3.05	5.4	3.82 to 7.8
45 to 55	1.01	0.76 to 1.33	2.53	1.99 to 3.2	6.57	4.62 to 9.56
55 to 69	1.26	0.96 to 1.67	2.66	2.07 to 3.4	6.78	4.72 to 9.93
Level of education						
No education	1 (ref)		1 (ref)		1 (ref)	
Primary education	0.76	0.64 to 0.9	1.21	1.05 to 1.39	1.8	1.49 to 2.17
Secondary education	0.88	0.7 to 1.11	1.72	1.45 to 2.04	2.89	2.33 to 3.6
Tertiary education	0.94	0.64 to 1.36	2.07	1.63 to 2.63	3.77	2.77 to 5.11
Employment status						
Employee	1 (ref)		1 (ref)		1 (ref)	
Self-employed	1.48	1.12 to 1.97	0.84	0.71 to 1.01	0.77	0.61 to 0.97
Student voluntary	1.82	1.33 to 2.5	0.59	0.46 to 0.76	0.52	0.37 to 0.72
Unemployed/retired	1.53	1.16 to 2.05	0.77	0.64 to 0.92	0.73	0.58 to 0.91
Physical activity						
≥150 min/week moderate or ≥75 min/week vigorous or ≥150 combination	1 (ref)		1 (ref)		1 (ref)	
1–149 min/week moderate or 1–74 min/week vigorous or 1–149 min/week combination	1.2	0.97 to 1.47	1.07	0.91 to 1.25	1.27	1.04 to 1.55
None	1.19	0.98 to 1.44	1.06	0.91 to 1.23	1.27	1.05 to 1.54
Fruit and vegetable consumption						
4–5 servings per day	1 (ref)		1 (ref)		1 (ref)	
2–3 servings per day	0.88	0.75 to 1.03	1.14	1.01 to 1.28	1.19	1.02 to 1.37
0–1 serving per day	1.15	0.92 to 1.44	1.01	0.85 to 1.2	1.36	1.1 to 1.68
Smoking						
Never or quit >12 months	1 (ref)		1 (ref)		1 (ref)	
Former, quit ≤12 months	1.53	0.62 to 3.4	0.13	0.02 to 0.52	0.86	0.27 to 2.3
Current	1.81	1.47 to 2.22	0.62	0.51 to 0.77	0.63	0.46 to 0.84
Alcohol consumption						
Never or quit >12 months	1 (ref)		1 (ref)		1 (ref)	
Current drinker	0.96	0.8 to 1.14	1.04	0.91 to 1.2	1	0.83 to 1.2
Heavy episodic drink	0.98	0.79 to 1.21	1.03	0.86 to 1.23	1.08	0.85 to 1.37

OR and their 95% CI were estimated by hierarchical multinomial mixed-effects regression using normal weight as the reference category and including a random effect on the country and year; they were adjusted for the variables listed in the table. The analysis was conducted in n=149 404 individuals without missing covariates.

#### Exploratory analysis

In exploratory analysis, adding contextual factors to the model did not change the association of individual risk factors with overweight or obesity as none of the contextual risk factors was significantly related to adverse BMI categories (online supplemental table 5). The distribution of the population characteristics by rural-urban status is reported in online supplemental table 6 and online supplemental figure 5. It shows in particular that underweight was overrepresented in rural areas, whereas overweight and obesity were overrepresented in urban areas. This was confirmed in multivariable analysis for overweight and obesity but not for underweight (online supplemental table 7).

As shown in online supplemental table 8, the PAF for grade 1 and grade 2 hypertension associated with overweight was, respectively, 11.99% and 19.45%; the corresponding PAF due to obesity was 18.55% and 37.07%. Similarly, the PAF for pre-diabetes associated with overweight and obesity was 4.88% and 9.46%; the corresponding PAF for type 2 diabetes was 13.33% and 26.11%, respectively (online supplemental table 9).

## Discussion

The analysis of 54 nationally/regionally representative and cross-sectional STEPS surveys from 2003 to 2022 across 36 countries from the WHO African region reveals three main findings. Almost 30% (ie, 26%) were overweight or obese, with females being much more affected than males. Furthermore, the 20 years trend analysis reveals a significant increase in obesity and underweight rates, and a plateau for overweight. This underscores the double burden of malnutrition in the region. Strikingly, the most educated were more likely to have overweight or obesity than the less educated.

The NCD-RisC has provided regular data on BMI worldwide both in children and in adults.^4 7 8 30^ In the latest analysis, the prevalence of obesity in 2022 ranged from <20% (Rwanda) up to 50% (South Africa) in African adult women and from 10% (Sierra Leone) to 30% (Central African Republic) in adult men, respectively.^8^ By combining hundreds of data sources, these studies were by far the most extensive and exhaustive. Despite rigorous statistical approaches, the use of multiple and heterogeneous data sources may, however, raise some concerns. Furthermore, six multi-country studies using data from demographic and health surveys have examined these questions specifically in Africa.^931^,^35^ In the largest study comprising 24 studies, in 2014, the prevalence of overweight ranged from 23.1% (Zambia) to 44% (Egypt) and that of obesity from 11.5% (Zambia) to 39.2% (Egypt).^15^ However, this study focused on females aged 15 to 49 years living in urban areas only.

The lower rates of overweight and obesity reported here, respectively 18.1% and 9.2%, have several explanations. First, these rates reflect the situation of the WHO African region only and not of the entire African continent. In particular, data from Northern Africa, which, with the exception of Algeria, are not part of the WHO African region, and data from South Africa, which is part of the WHO African region, were not available, although these are places where overweight and obesity rates are the most explosive. Also, the reliance on one sole data source (STEPs surveys) and the use of standardised data collection and measures needs to be considered.

In contrast to patterns observed in the Global North—where men often show similar or higher prevalence of overweight and obesity, and higher education is related to lower risk of overweight^36 37^—the current study revealed higher prevalence rates in women and in the most educated, likely reflecting contextual differences in lifestyle, socioeconomic transitions and cultural perceptions of body size. The substantial sex disparities in the prevalence of overweight and obesity are consistent with the results of previous studies in Africa.^915^,^17^ This can be attributed to socio-cultural norms that often prioritise larger body sizes for women, influencing their dietary habits and physical activity levels.^38^,^40^ Additionally, economic and healthcare-related factors, such as limited access to health prevention services including nutrition education, may further widen these gaps. Educational attainment could also play a role; however, it varies across settings and should be interpreted within each country’s specific context. Similarly, previous studies conducted in LMICs and in Africa have reported a positive association between higher education and overweight and obesity.^4 9 15 17 35 41^ In LMICs, higher education correlates with elevated socioeconomic status and access to Western diets and sedentary lifestyles, contributing to higher obesity rates. Furthermore, the correlation between education and overweight/obesity is influenced by multiple factors, fluctuates over time and is intricately linked to evolving dietary patterns across different countries.^42^ Additionally, the less educated are likely to engage in manual labour thus increasing physical activity coupled with undernutrition, which limits weight gain. Taken together, these findings underscore the need for targeted public health interventions that consider the unique socio-cultural and economic contexts affecting women in the WHO African region. Also, the results raise the point that education and awareness campaigns should not solely target individuals with lower education levels, as those with higher education may also require information and support.

Studies in sub-Saharan Africa have shown clear rural–urban differences in BMI distribution, with underweight more common in rural areas, and overweight and obesity more prevalent in urban populations.^3543^,^47^ Our findings reflect this pattern both in unadjusted and multivariable analysis. This is consistent with the ongoing nutrition transition in urban settings with increased intake of processed, energy-dense foods, driven by higher income and food access in urban settings.^35 45 47 48^ Differences in economic development and urbanisation also shape food environments and lifestyle patterns across the WHO African region.^9 35^

The present study further underscores the dual burden of malnutrition in the African region, showing significant increased trends in both obesity and underweight. Interestingly, in their latest analysis, the NCD-RisC reports a significantly increasing trend in prevalence of obesity from 1990 to 2022 worldwide,^8^ and the largest increases were seen in several sub-Saharan African countries. Furthermore, there were several sub-Saharan African countries where the underweight prevalence was above 10% in women in 1990 and that did not show any detectable decreasing trend.^8^ This is in contrast with what is seen in most of the remaining countries. This further underscores the critical need for interventions in sub-Saharan Africa that address both obesity and undernutrition. Contrary to the present study, the NCD-RisC reported a significant increase in mean BMI in Africa between 1980 and 2014, from 21.0 to 23.0 kg/m² in males and from 21.9 to 24.9 kg/m² in females, respectively.^6^ One possible reason for this apparent discrepancy is that the earlier and longer period (1980–2014) in the NCD-RisC analysis may have captured the earlier stages of the nutrition transition. Interestingly, the mean levels of BMI in our study are consistent with the mean levels of BMI achieved in the late period of the NCD risk evaluation.^49^ Furthermore, the projections made by the 2025 World Obesity Federation Atlas, indicating that the number of adults with class II obesity (BMI ≥35) is expected to increase by over 200% between 2010 and 2030 (from 11.8 million to 37.2 million) in Africa, is consistent with the results of our trend analysis on the prevalence of obesity.^50^

The WHO is intensifying its efforts to address the rising burden of obesity by focusing on highly cost-effective ‘best buy’ interventions.^51 52^ These include advocating for the taxation of sugar-sweetened beverages, reducing the intake of harmful fats through initiatives like the REPLACE Trans Fat programme, and promoting policies to limit the harmful marketing of unhealthy foods and beverages, particularly to children.^53^ Additionally, WHO’s Global Action Plan for the Prevention and Control of NCDs and the Global Strategy on Diet, Physical Activity and Health emphasise creating environments that support healthy diets and physical activity.^54^ Our findings indicate that these efforts should be tailored according to sex and gender, but also according to the socioeconomic background. Moreover, incorporating primordial prevention, focusing on preventing the emergence of risk factors such as poor diet and physical inactivity, could serve as a key complementary strategy.^55 56^

This study has some limitations. The study covered 36 out of the 47 WHO African region member states. In particular, data from South Africa, a country with one of the highest rates of overweight and obesity, were not available. Additional potential determinants of overweight and obesity, including cultural norms, accessibility to preventive healthcare services and the commercial determinants of health, were not studied.^44^ Similarly, and despite adjustment for individual education and GDP, residual confounding to direct household income or household expenditure cannot be excluded. Our dietary metric, based only on daily fruit and vegetable consumption, may misclassify adherence to healthy diets as it does not capture total energy intake, nutrient composition or validated dietary indices. The survey focused on adults aged 18–69, excluding children and older adults. Despite being the most widely used and standardised indicator for population-based surveillance of overweight and obesity, BMI is a marker of overall adiposity but does not address fat distribution and abdominal obesity in particular.^57 58^

## Conclusion

This extensive analysis conducted specifically in the WHO African region highlights that obesity and overweight in adults continue to be a health priority. These findings support the urgent need to intensify preventive health policies and programmes in the WHO African region.

## Supplementary material

10.1136/bmjgh-2025-019988 online supplemental file 1

## Data Availability

Data are available upon reasonable request.
