# Supplementary material for: Prevalence, time trends and associated factors of adult overweight and obesity in 36 countries in the WHO African region from 2003 to 2022: a study of 54 WHO STEPS surveys representing 156 million adults
Source: BMJ Glob Health. 2026 Jan 13;11(1):e019988. doi: 10.1136/bmjgh-2025-019988 (PMC12820823; doi:10.1136/bmjgh-2025-019988)
Supplement: online supplemental file 1 [file bmjgh-11-1-s001.docx]

**Supplementary appendix**

**Supplement to the paper on: Prevalence, time-trends and associated factors of adult overweight or obesity in 36 countries in the WHO African region from 2003 to 2022: a study of 54 WHO STEPS Surveys representing 156 million Adults.**

Table of Contents

[Appendix 1: Data sources 4](#_Toc208427120)

[Steps for Calculating Sampling Weights in WHO STEPS: 5](#_Toc208427121)

[Appendix 2: Definition Tobacco Use and Alcohol Consumption 6](#_Toc208427122)

[Appendix 3: Spatio temporal model estimating trends in BMI and BMI categories across countries and over years 6](#_Toc208427123)

[Spatial and spatio-temporal models with R-INLA 6](#_Toc208427124)

[Data Preprocessing 7](#_Toc208427125)

[Appendix 4: Tables 8](#_Toc208427126)

[Supplementary Table 1: Characteristics of individual WHO STEP survey in the African region by country and by year from 2003 to 2022 8](#_Toc208427127)

[Supplementary Table 2. Unweighted prevalences of BMI categories 10](#_Toc208427128)

[Supplementary Table 3. Additional characteristics of study participants overall and by BMI categories. 11](#_Toc208427129)

[Supplementary Table 4: Sex stratified analysis of factors associated with overweight, and obese status as compared to normal weight 13](#_Toc208427130)

[Supplementary Table 5: Factors associated with overweight, and obese status as compared to normal weight including contextual factors 15](#_Toc208427131)

[Supplementary Table 6. Characteristics of study participants overall and by Urban and Rural Residence 18](#_Toc208427132)

[Supplementary Table 7. Factors associated with overweight, and obese status as compared to normal weight further adjusted for rural/urban status (N=111577). 22](#_Toc208427133)

[Supplementary Table 8. Population Attributable Risk of high blood pressure due to abnormal BMI Categories 24](#_Toc208427134)

[Supplementary Table 9. Population Attributable Risk of High Blood Sugar due to abnormal BMI Categories 25](#_Toc208427135)

[Appendix 5: Figures 26](#_Toc208427136)

[Supplementary Figure 1: Flowchart of the study. 26](#_Toc208427137)

[Supplementary Figure 2. Map of African countries with available WHO STEPS Surveys included in the study (2003–2022) 27](#_Toc208427138)

[Supplementary Figure 3. Distribution of BMI categories by study periods (2003–2022) 28](#_Toc208427139)

[Supplementary Figure 4. Distribution of BMI categories by sub regions (2003–2022) 28](#_Toc208427140)

[Supplementary Figure 5. Distribution of BMI categories overall and by Urban and Rural Residence (N=111577) 29](#_Toc208427141)

[Supplementary Figure 6. Trend in age-standardised prevalence of Overweight overall and by sex from 2003 – 2022. Imputed data for all the 47 countries in the WHO African region 30](#_Toc208427142)

[Supplementary Figure 7. Trend in age-standardised prevalence of underweight, overall and by sex from 2003 – 2022. Imputed data for all the 47 countries in the WHO African region 31](#_Toc208427143)

[Supplementary Figure 8. Distribution of BMI categories by country 34](#_Toc208427144)

[Supplementary Figure 9: Estimated age-standardised mean BMI in women by country in 2003 and 2022 35](#_Toc208427145)

[Supplementary Figure 10: Estimated age-standardised mean BMI in men by country in 2003 and 2022 36](#_Toc208427146)

[Supplementary Figure 11. Estimated age-standardized mean BMI by country and by year from 2003 to 2022 37](#_Toc208427147)

[Supplementary Figure 12. Estimated age-standardized mean BMI in women by country and by year from 2003 to 2022 38](#_Toc208427148)

[Supplementary Figure 13. Estimated age-standardized mean BMI in men by country and by year from 2003 to 2022 39](#_Toc208427149)

[Supplementary Figure 14. Estimated age-standardized prevalence of obesity by country from 2003 to 2022 40](#_Toc208427150)

[Supplementary Figure 15. Estimated age-standardized prevalence of obesity in women by country from 2003 to 2022 41](#_Toc208427151)

[Supplementary Figure 16. Estimated age-standardized prevalence of obesity in men by country from 2003 to 2022 42](#_Toc208427152)

[Supplementary Figure 17. Trend in age-standardised mean BMI from 2003 – 2022 for males. Imputed data for all the 47 countries in the WHO African region 43](#_Toc208427153)

[Supplementary Figure 18. Trend in age-standardised mean BMI from 2003 – 2022 for females. Imputed data for all the 47 countries in the WHO African region 44](#_Toc208427154)

[References: 45](#_Toc208427155)

# **Appendix 1:** Data sources

We used data from the population-based, subnational/national [World Health Organization (WHO) STEPwise](https://www.who.int/teams/noncommunicable-diseases/surveillance/systems-tools/steps) surveys from 2003 to 2022. We requested each eligible STEPS dataset from a list of these surveys that the WHO maintains in the NCD Microdata Repository. We were granted access to these data, which are available in the WHO STEPS Survey repository.

The WHO STEPwise approach to surveillance (STEPS) is a standardized method for collecting, analyzing, and disseminating data on non-communicable diseases (NCDs) and their risk factors. It provides countries with a framework to monitor the prevalence of NCDs and track progress towards national and global health goals. (1)

The WHO STEPS implementation leverages a standardized and consistent methodology to ensure robust data collection and analysis across countries. This comprehensive approach begins with meticulous planning and preparation, including stakeholder engagement with government bodies and health institutions to secure collaboration, training field staff on standardized data collection and ethical procedures, and conducting pilot tests to refine methodologies. During the data collection phase, household surveys gather information from representative samples, with rigorous quality control measures like repeat measurements and cross-checking to guarantee data accuracy. Statistical analysis of the collected data identifies prevalence rates, risk factor correlations, and trends, which are then detailed in comprehensive reports for policymakers, health professionals, and the public.

Here’s a detailed look at the WHO STEPwise approach:

The STEPS has three main objectives:

- Standardization: To provide a common methodology for countries to collect and compare NCD data.
- Flexibility: To allow countries to use the approach according to their resources and needs.
- Scalability: To enable countries to start with basic data collection and expand as resources allow.

The survey is conducted in three steps, each building on the previous one to provide a comprehensive overview of NCDs and their risk factors: Step 1: A questionnaire-based interview to collect information on demographic characteristics and risk factors for NCDs; Step 2: Physical measurements; and Step 3: Biochemical measurements.

- Step 1: Questionnaire:
- Demographic Information: Collects data on age, sex, education, and other sociodemographic variables.
- Behavioural Risk Factors: Includes questions on tobacco use, alcohol consumption, diet, and physical activity, collected in face to face.
- History of Disease: Gathers information on known history of diseases such as hypertension, diabetes, and cardiovascular conditions.
- Step 2: Physical Measurements:
- Anthropometric Data: Measures height, weight, and waist circumference to assess obesity and overweight.
- Blood Pressure: Records systolic and diastolic blood pressure to identify hypertension.
- Step 3: Biochemical Measurements
- Blood Samples: to measure blood glucose levels and lipid profiles (cholesterol, triglycerides) on fresh samples (?).
- Urine Samples: May include urine tests for additional markers relevant to NCDs.

In the **WHO STEPS** survey, sampling weights are calculated to ensure that the data collected are representative of the target population. The sampling weight corrects for unequal probabilities of selection and adjusts for non-response and post-stratification. Here’s a breakdown of how the sampling weight is typically calculated in the WHO STEPS survey:

## **Steps for Calculating Sampling Weights in WHO STEPS:**

1. **Design Weight (Inverse of Selection Probability):**
   - The **design weight** is calculated as the inverse of the probability that a participant was selected. This accounts for the different probabilities of selection due to the sampling design, which often includes stratification, clustering, and multistage sampling.
   - **Formula:** Design Weight (DW)=1PiDesign Weight (DW)=Pi​1​ where PiPi​ is the probability of selecting individual ii.

In a multistage design, the overall probability of selection is the product of the selection probabilities at each stage (e.g., selecting a household, then an individual within the household).

1. **Non-Response Adjustment:**
   - Non-response weights adjust for differences in response rates across different groups. The aim is to correct for any bias introduced by non-responders.
   - This is done by adjusting the design weight based on the response rate in each stratum or cluster.
   - **Formula:** Non Response Weight (NRW)=Number of eligible individualsNumber of respondentsNon-Response Weight (NRW)=Number of respondentsNumber of eligible individuals​
2. **Post-Stratification Weight:**
   - After adjusting for the sampling design and non-response, a **post-stratification weight** is often applied to ensure the final weighted data matches known population distributions (e.g., by age, sex, or region) based on census or population estimates.
   - This helps adjust for any discrepancies between the sample and the population.
   - **Formula:** Post-Stratification Weight (PSW)=Population proportionSample proportionPost-Stratification Weight (PSW)=Sample proportionPopulation proportion​
3. **Final Sampling Weight:**
   - The **final sampling weight** is the product of the design weight, non-response adjustment, and post-stratification adjustment.
   - **Formula:**Final Weight (FW)=DW×NRW×PSWFinal Weight (FW)=DW×NRW×PSW

# **Appendix 2:** Definition Tobacco Use and Alcohol Consumption

**Tobacco use** is classified into three categories based on smoking history and current use:

- Never or quit >12 months: Individuals who have never smoked or who quit smoking more than 12 months ago.
- Former, quit ≤12 months: Individuals who previously smoked but quit within the past 12 months.
- Current: Individuals who currently smoke.

**Alcohol Consumption**: Alcohol consumption is categorized based on drinking frequency and intensity:

- Never or quit >12 months: Individuals who have never consumed alcohol or who quit drinking more than 12 months ago.
- Current drinker: Individuals who currently consume alcohol, non-heavy drinkers
- Heavy episodic drinker: Individuals who engage in heavy episodic drinking, defined as consuming a large amount of alcohol on a single occasion (Six or more drinks within a short period).

# **Appendix 3:** Spatio temporal model estimating trends in BMI and BMI categories across countries and over years

## **Spatial and spatio-temporal models with R-INLA**

The model utilizes a multivariate regression framework to estimate body mass index (BMI) and its categories (Normal, Underweight, Overweight, Obesity) across different countries and years, from 2003 to 2022, incorporating both spatial (latitude and longitude) and temporal (year) covariates. BMI values were not only estimated for missing years and/or countries within the initially studied 36 countries, but also, the spatiotemporal analysis enabled us to extend BMI prevalence estimates to the 11 African countries for which we were not granted STEPS data, thereby providing comprehensive trends for all 47 member states in the African region

## **Data Preprocessing**

To ensure consistency and comparability, numeric UNDP and world bank covariates were normalized, and missing values were imputed using the mean of the available data. Covariates that significantly influenced BMI were identified and selected based on a backward elimination process using the Akaike Information Criterion (AIC).The World Bank and UNDP covariates include the Food Production Index, GDP per capita, grams of fat per day per capita, prevalence of undernourishment, employment in agriculture, value added in agriculture, and urban population.^18,19^ The model uses the Integrated Nested Laplace Approximation (INLA) approach, which is computationally efficient and provides accurate approximations for Bayesian inference.^16–18^ This method allows for the inclusion of complex random effects structures to capture spatial and temporal dependencies. By normalizing numeric covariates and imputing missing values, the model ensures data consistency and reliability. The Besag-York-Mollié (BYM) model accounts for structured and unstructured spatial variation, while independent and identically distributed random effects handle temporal components.^21^ INLA's flexibility, efficiency, and robustness make it well-suited for analysing the spatial and temporal trends in BMI, providing reliable parameter estimates and uncertainty quantification.^22^ This comprehensive approach leverages the strengths of Bayesian modelling, enabling detailed analysis of BMI data across different countries and periods. All estimations were provided by 95% credible interval.

# **Appendix 4: Tables**

## Supplementary Table 1: Characteristics of individual WHO STEP survey in the African region by country and by year from 2003 to 2022

|  | **Country** | **WHO AFRO Sub-region** | **Year of survey** | **Rural, urban or both** | **Country income level** | **Sample size** |
| --- | --- | --- | --- | --- | --- | --- |
| 1 | Algeria | North Africa | 2003 | Both | Lower_Middle | 4047 |
| 2 | Algeria | North Africa | 2016 | Urban | Upper_Middle | 6628 |
| 3 | Benin | West Africa | 2007 | Both | Low | 2522 |
| 4 | Benin | West Africa | 2008 | Both | Low | 6795 |
| 5 | Benin | West Africa | 2015 | Rural | Low | 4847 |
| 6 | Botswana | South Africa | 2007 | Rural | Upper_Middle | 3820 |
| 7 | Botswana | South Africa | 2014 | Both | Upper_Middle | 3738 |
| 8 | Burkina Faso | West Africa | 2013 | Rural | Low | 4475 |
| 9 | Cabo Verde | West Africa | 2007 | Rural | Lower_Middle | 1724 |
| 10 | Cabo Verde | West Africa | 2020 | Rural | Lower_Middle | 4458 |
| 11 | Cameroon | Central Africa | 2003 | Urban | Low | 8019 |
| 12 | Central African Republic | Central Africa | 2010 | Both | Low | 3812 |
| 13 | Central African Republic | Central Africa | 2017 | Both | Low | 3268 |
| 14 | Chad | Central Africa | 2008 | Urban | Low | 1840 |
| 15 | Comoros | East Africa | 2011 | Rural | Low | 5042 |
| 16 | Congo | Central Africa | 2004 | Urban | Low | 1967 |
| 17 | Côte d’Ivoire | West Africa | 2005 | Both | Low | 4169 |
| 18 | Democratic Republic of the Congo | Central Africa | 2005 | Urban | Low | 1658 |
| 19 | Eritrea | East Africa | 2004 | Both | Low | 2067 |
| 20 | Eritrea | East Africa | 2010 | Both | Low | 5653 |
| 21 | Eswatini | South Africa | 2007 | Both | Lower_Middle | 1259 |
| 22 | Eswatini | South Africa | 2014 | Both | Lower_Middle | 2835 |
| 23 | Ethiopia | East Africa | 2006 | Both | Low | 3952 |
| 24 | Ethiopia | East Africa | 2015 | Rural | Low | 8749 |
| 25 | Gabon | Central Africa | 2009 | Urban | Upper_Middle | 2455 |
| 26 | Gambia | West Africa | 2010 | Urban | Low | 3529 |
| 27 | Ghana | West Africa | 2006 | Urban | Low | 2479 |
| 28 | Guinea | West Africa | 2009 | Both | Low | 2164 |
| 29 | Kenya | East Africa | 2015 | Rural | Lower_Middle | 4266 |
| 30 | Lesotho | South Africa | 2012 | Both | Lower_Middle | 2170 |
| 31 | Liberia | West Africa | 2011 | Both | Low | 2269 |
| 32 | Liberia | West Africa | 2022 | Both | Low | 3892 |
| 33 | Madagascar | East Africa | 2005 | Both | Low | 5068 |
| 34 | Malawi | South Africa | 2009 | Rural | Low | 4844 |
| 35 | Malawi | South Africa | 2017 | Rural | Low | 4012 |
| 36 | Mali | West Africa | 2007 | Urban | Low | 2243 |
| 37 | Mali | West Africa | 2013 | Both | Low | 1759 |
| 38 | Mauritania | West Africa | 2006 | Urban | Low | 2283 |
| 39 | Mozambique | Central Africa | 2005 | Both | Low | 2960 |
| 40 | Niger | West Africa | 2007 | Both | Low | 2487 |
| 41 | Niger | West Africa | 2021 | Both | Low | 5329 |
| 42 | Rwanda | East Africa | 2012 | Both | Low | 6482 |
| 43 | Rwanda | East Africa | 2021 | Rural | Low | 5509 |
| 44 | Sao Tome and Principe | Central Africa | 2008 | Both | Lower_Middle | 2286 |
| 45 | Sao Tome and Principe | Central Africa | 2019 | Urban | Lower_Middle | 2291 |
| 46 | Seychelles | East Africa | 2004 | Both | Upper_Middle | 1255 |
| 47 | Sierra Leone | West Africa | 2008 | Rural | Low | 4525 |
| 48 | Togo | West Africa | 2010 | Both | Low | 3858 |
| 49 | Togo | West Africa | 2021 | Both | Low | 3750 |
| 50 | Uganda | East Africa | 2014 | Urban | Low | 3682 |
| 51 | United Republic of Tanzania | East Africa | 2011 | Urban | Low | 2525 |
| 52 | United Republic of Tanzania | East Africa | 2012 | Rural | Low | 5402 |
| 53 | Zambia | South Africa | 2008 | Urban | Low | 1779 |
| 54 | Zambia | South Africa | 2017 | Rural | Lower_Middle | 4004 |

## Supplementary Table 2. Unweighted prevalences of BMI categories

| **Characteristic** | **Body Mass Index** | | | |
| --- | --- | --- | --- | --- |
|  | **Normal**  **(18•5 to 24·9 kg/m^2^)** | **Underweight**  **(< 18·5kg/m^2^)** | **Overweight**  **(25 to 29·9 kg/m^2^),** | **Obese**  **(>=30kg/m^2^)** |
| **Overall** | 114 560 (61,7%)**^1^** | 20 991 (11,5%)**^1^** | 40 470 (17,8%)**^1^** | 22 880 (9,0%)**^1^** |
| **Sexe** |  |  |  |  |
| Famales | 59 543 (55,6%) | 11 499 (10,3%) | 26 319 (20,8%) | 18 450 (13,3%) |
| Male | 55 017 (67,8%) | 9 492 (12,8%) | 14 151 (14,9%) | 4 430 (4,6%) |
| ^1^ Percentage in column. | | | | |

## Supplementary Table 3. Additional characteristics of study participants overall and by BMI categories.

| **Characteristic** |  | **Body Mass Index (kg/m^2^)** | | | | |
| --- | --- | --- | --- | --- | --- | --- |
|  |  | **Overall**,  N = 198901 (100%)*^1^* | **Underweight**  **(< 18**·**5kg/m^2^),**  N = 20991 (11·5%)*^2^* | **Normal**  **(18**·**5 to 24**·**9 kg/m^2^),**  N = 114560 (61·7%)*^2^* | **Overweight**  **(25 to 29**·**9 kg/m^2^),**  N = 40470 (17·8%)*^2^* | **Obese**  **(>=30kg/m^2^),**  N = 22 880  (9·0%)*^2^* |
| **Age groups** | **195 633** |  |  |  |  |  |
| [18;25) |  | 20 454 (18·6%) | 2 459 (14·5%) | 14 364 (71·3%) | 2 772 (11·0%) | 859 (3·2%) |
| [25;35) |  | 62 115 (32·0%) | 5 945 (9·9%) | 38 954 (66·3%) | 11 836 (16·8%) | 5 380 (7·0%) |
| [35;45) |  | 48 218 (22·8%) | 4 406 (10·0%) | 26 406 (57·5%) | 10 810 (20·8%) | 6 596 (11·8%) |
| [45;55) |  | 35 222 (15·0%) | 3 742 (11·8%) | 17 920 (52·9%) | 8 043 (21·6%) | 5 517 (13·7%) |
| [55;69] |  | 29 624 (11·7%) | 3 843 (14·0%) | 15 197 (53·0%) | 6 446 (21·0%) | 4 138 (12·0%) |
| **Residency** | **111 577** |  |  |  |  |  |
| Rural |  | 55 523 (61·3%) | 6 183 (14·0%) | 35 591 (67·9%) | 9 523 (12·9%) | 4 226 (5·3%) |
| Urban |  | 56 054 (38·7%) | 3 868 (7·5%) | 28 007 (50·3%) | 14 617 (26·6%) | 9 562 (15·6%) |
| **Marital Status** | **132 687** |  |  |  |  |  |
| Single |  | 28 590 (23·9%) | 2 590 (12·2%) | 16 650 (65·3%) | 5 950 (15·9%) | 3 400 (6·6%) |
| Married/Cohabitating |  | 86 816 (66·7%) | 9 553 (11·4%) | 51 205 (60·3%) | 16 857 (18·6%) | 9 201 (9·8%) |
| Divorced/Separated/Widowed |  | 17 281 (9·4%) | 2 382 (13·0%) | 9 376 (58·3%) | 3 275 (17·5%) | 2 248 (11·2%) |
| **Employment status** | **183 822** |  |  |  |  |  |
| Employee |  | 30 045 (16·6%) | 2 250 (6·1%) | 16 499 (57·6%) | 7 533 (25·4%) | 3 763 (11·0%) |
| Self-employed |  | 67 726 (31·1%) | 6 772 (9·4%) | 41 507 (64·6%) | 12 726 (18·0%) | 6 721 (8·0%) |
| Student/voluntary |  | 19 010 (16·7%) | 2 864 (19·3%) | 12 264 (70·1%) | 2 589 (8·0%) | 1 293 (2·7%) |
| Unemployed/retired |  | 67 041 (35·7%) | 8 037 (12·7%) | 36 617 (58·0%) | 13 564 (18·0%) | 8 823 (11·3%) |
| **BP category, mmHg** | **193 183** |  |  |  |  |  |
| Normal BP (<130/85 mmHg) |  | 109 598 (60·2%) | 13 835 (13·7%) | 68 067 (65·1%) | 19 072 (14·9%) | 8 624 (6·2%) |
| High-normal (130–139/85–89 mmHg) |  | 36 584 (19·3%) | 3 098 (9·4%) | 20 544 (59·0%) | 8 282 (20·9%) | 4 660 (10·7%) |
| Grade 1 hypertension (140–159/90–99 mmHg) |  | 27 491 (12·9%) | 2 202 (7·6%) | 14 309 (56·4%) | 6 591 (22·5%) | 4 389 (13·4%) |
| Grade 2 hypertension (≥160/100 mmHg) |  | 19 510 (7·6%) | 1 449 (7·9%) | 8 737 (50·3%) | 5 178 (24·1%) | 4 146 (17·7%) |
| Mean (SD); ^1^ Percentage in column (%); ^2^ Percentage in row. All percentages are weighted; all n are unweighted | | | | | | |

## Supplementary Table 4: Sex stratified analysis of factors associated with overweight, and obese status as compared to normal weight

| **Predictors** | **Underweight** | | | | **Overweight** | | | | **Obese** | | | | |
| --- | --- | --- | --- | --- | --- | --- | --- | --- | --- | --- | --- | --- | --- |
|  | **Women** | | **Men** | | **Women** | | **Men** | | **Women** | | **Men** | | |
|  | **OR** | **95% CI** | **OR** | **95% CI** | **OR** | **95% CI** | **OR** | **95% CI** | **OR** | **95% CI** | **OR** | **95% CI** |  |
| **Age groups** |  |  |  |  |  |  |  |  |  |  |  |  |  |
| [18;25) | 1(ref) |  | 1(ref) |  | 1(ref) |  | 1(ref) |  | 1(ref) |  | 1(ref) |  |  |
| [25;35) | 0.96 | 0.71 – 0.88 | 0.69 | 0.64 – 0.75 | 1.80 | 1.69 – 1.91 | 2.09 | 1.91 – 2.29 | 3.11 | 2.85 – 3.40 | 2.79 | 2.32 – 3.35 |  |
| [35;45) | 0.89 | 0.78 – 0.91 | 0.75 | 0.69 – 0.82 | 2.48 | 2.34 – 2.64 | 3.22 | 2.94 – 3.53 | 6.30 | 5.77 – 6.88 | 5.78 | 4.82 – 6.93 |  |
| [45;55) | 0.96 | 0.89 – 1.05 | 0.95 | 0.87 – 1.04 | 2.75 | 2.58 – 2.94 | 3.79 | 3.45 – 4.16 | 8.45 | 7.71 – 9.25 | 7.20 | 5.99 – 8.67 |  |
| [55;69] | 1.06 | 1.03 – 1.23 | 1.13 | 1.03 – 1.24 | 2.69 | 2.51 – 2.88 | 3.69 | 3.35 – 4.06 | 7.80 | 7.10 – 8.58 | 7.33 | 6.07 – 8.85 |  |
| **Level of education** |  |  |  |  |  |  |  |  |  |  |  |  |  |
| No education | 1(ref) |  | 1(ref) |  | 1(ref) |  | 1(ref) |  | 1(ref) |  | 1(ref) |  |  |
| Primary education | 0.74 | 0.70 – 0.78 | 0.90 | 0.85 – 0.95 | 1.42 | 1.36 – 1.48 | 1.27 | 1.20 – 1.34 | 1.81 | 1.73 – 1.90 | 1.47 | 1.33 – 1.63 |  |
| Secondary education | 0.70 | 0.66 – 0.76 | 0.76 | 0.71 – 0.82 | 1.78 | 1.70 – 1.87 | 1.80 | 1.70 – 1.92 | 2.51 | 2.37 – 2.66 | 2.43 | 2.19 – 2.70 |  |
| Tertiary education | 0.69 | 0.61 – 0.79 | 0.69 | 0.62 – 0.77 | 1.89 | 1.75 – 2.04 | 2.49 | 2.31 – 2.69 | 2.80 | 2.57 – 3.05 | 4.01 | 3.56 – 4.52 |  |
| **Physical activity** |  |  |  |  |  |  |  |  |  |  |  |  |  |
| ≥150 min/wk moderate or ≥75 min/wk vigorous or ≥150 combination) | 1(ref) |  | 1(ref) |  | 1(ref) |  | 1(ref) |  | 1(ref) |  | 1(ref) |  |  |
| 1–149 min/wk moderate or 1–74 min/wk vigorous or 1–149 min/wk combination) | 1.07 | 1.01 – 1.15 | 1.07 | 0.99 – 1.15 | 1.09 | 1.04 – 1.15 | 1.11 | 1.05 – 1.18 | 1.21 | 1.14 – 1.28 | 1.21 | 1.10 – 1.34 |  |
| None | 1.03 | 0.96 – 1.10 | 1.14 | 1.07 – 1.22 | 1.06 | 1.02 – 1.11 | 1.18 | 1.12 – 1.25 | 1.26 | 1.19 – 1.33 | 1.41 | 1.29 – 1.54 |  |
| **Fruit & vegetable consumption** |  |  |  |  |  |  |  |  |  |  |  |  |  |
| 4–5 servings per day | 1(ref) |  | 1(ref) |  | 1(ref) |  | 1(ref) |  | 1(ref) |  | 1(ref) |  |  |
| 2–3 servings per day | 0.98 | 0.92 – 1.03 | 0.91 | 0.86 – 0.97 | 1.08 | 1.04 – 1.12 | 1.09 | 1.04 – 1.14 | 1.12 | 1.07 – 1.17 | 1.16 | 1.07 – 1.25 |  |
| 0–1 serving per day | 1.04 | 0.96 – 1.13 | 0.91 | 0.84 – 0.99 | 1.09 | 1.04 – 1.16 | 1.00 | 0.94 – 1.07 | 1.19 | 1.12 – 1.27 | 1.08 | 0.97 – 1.21 |  |
| **Smoking** |  |  |  |  |  |  |  |  |  |  |  |  |  |
| Never or quit > 12 m | 1(ref) |  | 1(ref) |  | 1(ref) |  | 1(ref) |  | 1(ref) |  | 1(ref) |  |  |
| Former, quit ≤12 m | 1·63 | 0·81 - 3·02 | 1·63 | 0·81 - 3·02 | 0·51 | 0·25 - 0·96 | 0·51 | 0·25 - 0·96 | 0·31 | 0·10 - 0·79 | 0·31 | 0·10 - 0·78 |  |
| Current | 1·56 | 1·34 - 1·81 | 1·56 | 1·34 - 1·81 | 0·50 | 0·43 - 0·99 | 0·50 | 0·43 - 0·99 | 0·34 | 0·27 - 1·00 | 0·44 | 0·27 - 1·01 |  |
| **Alcohol consumption** |  |  |  |  |  |  |  |  |  |  |  |  |  |
| Never or quit > 12 m | 1(ref) |  | 1(ref) |  | 1(ref) |  | 1(ref) |  | 1(ref) |  | 1(ref) |  |  |
| Current drinker | 0·98 | 0·85 - 1·12 | 0·98 | 0·98 - 1·12 | 0·94 | 0·83 - 1·04 | 0·93 | 0·83 - 1·04 | 0·90 | 0·79 - 1·04 | 0·91 | 0·79 - 1·04 |  |
| Heavy episodic drink | 0·88 | 0·73 - 1·07 | 0·88 | 0·72 - 1·07 | 0·82 | 0·70 - 0·96 | 0·82 | 0·70 - 0·96 | 0·74 | 0·59 - 0·92 | 0·75 | 0·59 - 0·92 |  |
| OR: Odds Ratio, CI = Confidence Interval. Data are in OR (95% CI).  OR and their 95% CI were estimated hierarchical multinomial mixed-effects regression using normal weight as the reference category and including a random effect on the country and years; they were adjusted for the variables listed in the Table. | | | | | | | | | | | | | |

## Supplementary Table 5: Factors associated with overweight, and obese status as compared to normal weight including contextual factors

|  | **Underweight** | | | **Overweight** | | | **Obesity** | | |
| --- | --- | --- | --- | --- | --- | --- | --- | --- | --- |
| **Variable** | **OR** | **CI_lower** | **CI_upper** | **OR** | **CI_lower** | **CI_upper** | **OR** | **CI_lower** | **CI_upper** |
| **Sex** |  |  |  |  |  |  |  |  |  |
| Women | 1(ref) |  |  | 1(ref) |  |  | 1(ref) |  |  |
| Men | 1.04 | 0.94 | 1.15 | 0.52 | 0.48 | 0.56 | 0.21 | 0.19 | 0.24 |
| **Age Groups** |  |  |  |  |  |  |  |  |  |
| [18,25) | 1(ref) |  |  | 1(ref) |  |  | 1(ref) |  |  |
| [25,35) | 0.74 | 0.70 | 0.79 | 1.93 | 1.82 | 2.04 | 2.96 | 2.70 | 3.24 |
| [35,45) | 0.80 | 0.75 | 0.86 | 2.74 | 2.60 | 2.91 | 6.03 | 5.51 | 6.59 |
| [45,55) | 0.98 | 0.92 | 1.06 | 3.10 | 2.92 | 3.29 | 7.92 | 7.20 | 8.69 |
| [55,69] | 1.16 | 1.08 | 1.24 | 3.04 | 2.86 | 3.24 | 7.51 | 6.82 | 8.28 |
| **Level of Education** |  |  |  |  |  |  |  |  |  |
| No education | 1(ref) |  |  | 1(ref) |  |  | 1(ref) |  |  |
| Primary education | 0.82 | 0.78 | 0.85 | 1.33 | 1.28 | 1.38 | 1.65 | 1.58 | 1.73 |
| Secondary education | 0.76 | 0.72 | 0.81 | 1.72 | 1.65 | 1.80 | 2.38 | 2.25 | 2.51 |
| Tertiary education | 0.68 | 0.62 | 0.75 | 2.10 | 1.97 | 2.22 | 3.09 | 2.87 | 3.34 |
| **Employment status** |  |  |  |  |  |  |  |  |  |
| Employee |  |  |  |  |  |  |  |  |  |
| Self-employed | 1.48 | 1.12 | 1.97 | 0.88 | 0.73 | 1.05 | 0.82 | 0.65 | 1.03 |
| Student voluntary | 1.79 | 1.31 | 2.47 | 0.62 | 0.48 | 0.8 | 0.55 | 0.39 | 0.78 |
| Employee | 1.53 | 1.15 | 2.04 | 0.78 | 0.65 | 0.94 | 0.75 | 0.59 | 0.94 |
| **Physical activity** |  |  |  |  |  |  |  |  |  |
| ≥150 min/wk moderate or ≥75 min/wk vigorous or ≥150 combination | 1(ref) |  |  | 1(ref) |  |  | 1(ref) |  |  |
| 1–149 min/wk moderate or 1–74 min/wk vigorous or 1–149 min/wk combination | 1.05 | 0.99 | 1.11 | 1.08 | 1.04 | 1.13 | 1.22 | 1.15 | 1.29 |
| None | 1.13 | 1.07 | 1.18 | 1.10 | 1.06 | 1.15 | 1.32 | 1.26 | 1.39 |
| **Fruit & vegetable consumption** |  |  |  |  |  |  |  |  |  |
| 4–5 servings per day | 1(ref) |  |  | 1(ref) |  |  | 1(ref) |  |  |
| 2–3 servings per day | 0.93 | 0.89 | 0.97 | 1.13 | 1.08 | 1.17 | 1.13 | 1.08 | 1.17 |
| 0–1 serving per day | 0.95 | 0.89 | 1.01 | 1.17 | 1.11 | 1.24 | 1.17 | 1.11 | 1.24 |
| **Life Expectancy at Birth (years)** | 1.01 | 0.99 | 1.03 | 0.99 | 0.98 | 1.01 | 0.98 | 0.96 | 1.01 |
| **Mean Years of Schooling (years)** | 1.05 | 1.02 | 1.09 | 1.02 | 0.99 | 1.05 | 0.98 | 0.95 | 1.02 |
| **Gender Development Index** | 0.38 | 0.05 | 2.55 | 9.7 | 1.64 | 62.43 | 10.02 | 0.49 | 214.34 |
| **Grams of fat per day per capita** | 0.99 | 0.98 | 1.00 | 1.01 | 0.99 | 1.02 | 1.01 | 0.99 | 1.023 |
| **Prevalence of undernourishment** | 2.28 | 0.60 | 8.10 | 7.14 | 1.83 | 28.90 | 111.09 | 11.26 | 1296.61 |
| **Employment in agriculture (% of employment)** | 1.06 | 0.81 | 1.40 | 0.95 | 0.76 | 1.17 | 1.15 | 0.82 | 1.59 |
| **Urban population (% of total population)** | 0.61 | 0.21 | 2.06 | 1.92 | 0.59 | 5.66 | 2.27 | 0.36 | 15.09 |
| **GDP per capita, PPP (constant 2017 international $)** | 1.20 | 0.97 | 1.50 | 0.88 | 0.66 | 1.14 | 1.55 | 0.97 | 2.43 |
| **Food production index (2014-2016 = 100)** | 0.99 | 0.99 | 1.00 | 0.99 | 0.98 | 1.00 | 1.00 | 0.99 | 1.01 |
| OR: Odds Ratio, CI = Confidence Interval. Data are in OR (95% CI).  OR and their 95% CI were estimated hierarchical multinomial mixed-effects regression using normal weight as the reference category and including a random effect on the country and years; they were adjusted for the variables listed in the Table. | | | | | | | | | |

## Supplementary Table 6. Characteristics of study participants overall and by Urban and Rural Residence

| **Characteristics** | **Overall,**  **N = 111577 (100%)^1^** | **Rural,**  **N = 55523 (61%)^1^** | **Urban,**  **N = 56054 (38,7%)^1^** |
| --- | --- | --- | --- |
| **Body Mass Index** |  |  |  |
| Normal | 63 598 (61,1%) | 35 591 (67,9%) | 28 007 (50,3%) |
| Underweight | 10 051 (11,5%) | 6 183 (14,0%) | 3 868 (7,5%) |
| Overweight | 24 140 (18,2%) | 9 523 (12,9%) | 14 617 (26,6%) |
| Obesity | 13 788 (9,3%) | 4 226 (5,3%) | 9 562 (15,6%) |
| **Sexe** |  |  |  |
| Women | 65 326 (49,1%) | 31 885 (47,5%) | 33 441 (51,7%) |
| Men | 46 251 (50,9%) | 23 638 (52,5%) | 22 613 (48,3%) |
| **Age groups** |  |  |  |
| [18,25) | 12 192 (20,0%) | 5 001 (19,9%) | 7 191 (20,3%) |
| [25,35) | 36 317 (32,1%) | 18 081 (32,5%) | 18 236 (31,6%) |
| [35,45) | 27 777 (22,4%) | 14 293 (22,8%) | 13 484 (21,7%) |
| [45,55) | 19 384 (14,3%) | 9 904 (13,9%) | 9 480 (15,0%) |
| [55,69] | 15 907 (11,1%) | 8 244 (11,0%) | 7 663 (11,3%) |
| **Level of education** |  |  |  |
| No education | 31 683 (24,2%) | 20 576 (30,9%) | 11 107 (13,6%) |
| Primary education | 42 795 (45,1%) | 22 014 (48,7%) | 20 781 (39,5%) |
| Secondary education | 28 515 (23,5%) | 10 387 (16,9%) | 18 128 (33,9%) |
| Tertiary education | 8 035 (7,2%) | 2 318 (3,5%) | 5 717 (13,0%) |
| **Marital Situation** |  |  |  |
| Single | 17 142 (23,6%) | 6 270 (18,1%) | 10 872 (32,6%) |
| Married/Cohabitating | 54 098 (67,1%) | 34 283 (72,3%) | 19 815 (58,4%) |
| Divorced/Separated/Widowed | 10 656 (9,4%) | 6 597 (9,6%) | 4 059 (9,0%) |
| Missing observations | 11 124 786 | 5 656 764 | 5 468 022 |
| **Employment status** |  |  |  |
| Employee | 19 322 (18,2%) | 8 588 (13,7%) | 10 734 (25,7%) |
| Self-employed | 37 373 (28,9%) | 22 796 (30,3%) | 14 577 (26,5%) |
| Student/voluntary | 11 525 (19,0%) | 6 804 (23,5%) | 4 721 (11,4%) |
| Unemployed/retired | 34 478 (34,0%) | 17 019 (32,5%) | 17 459 (36,4%) |
| **Physical activities** |  |  |  |
| ≥150 min/wk moderate or ≥75 min/wk vigorous or ≥150 combination | 43 389 (55,5%) | 22 555 (56,9%) | 20 834 (53,3%) |
| 1–149 min/wk moderate or 1–74 min/wk vigorous or 1–149 min/wk combination | 17 200 (18,3%) | 7 321 (17,4%) | 9 879 (19,8%) |
| None | 47 638 (26,2%) | 24 003 (25,8%) | 23 635 (26,9%) |
| **Fruit & vegetable consumption** |  |  |  |
| 4–5 Components | 45 953 (50,9%) | 25 584 (53,7%) | 20 369 (46,7%) |
| 2–3 Components | 32 719 (38,9%) | 17 403 (36,8%) | 15 316 (42,1%) |
| 0–1 Component | 10 214 (10,2%) | 4 980 (9,5%) | 5 234 (11,2%) |
| **Smoking** |  |  |  |
| Never or quit > 12 m | 94 981 (88,0%) | 48 780 (88,9%) | 46 201 (86,6%) |
| Former, quit ≤12 m | 708 (0,8%) | 338 (0,7%) | 370 (0,9%) |
| Current | 11 787 (11,2%) | 6 210 (10,5%) | 5 577 (12,4%) |
| Missing observations | 1 526 488 | 124 955 | 1 401 533 |
| **Alcohol consumption** |  |  |  |
| Never or quit > 12 m | 64 266 (62,0%) | 34 999 (59,9%) | 29 267 (65,5%) |
| Current user | 31 715 (25,5%) | 14 285 (26,2%) | 17 430 (24,3%) |
| Heavy episodic drink | 10 495 (12,5%) | 6 231 (13,9%) | 4 264 (10,3%) |
| **BP classification, mmHg** |  |  |  |
| Normal BP | 61 960 (61,4%) | 31 660 (62,1%) | 30 300 (60,3%) |
| High-normal BP | 20 316 (19,4%) | 10 768 (19,2%) | 9 548 (19,6%) |
| Grade 1 hypertension | 14 452 (12,6%) | 7 415 (12,6%) | 7 037 (12,6%) |
| Grade 2 hypertension | 9 843 (6,6%) | 4 598 (6,1%) | 5 245 (7,4%) |
| **Systolic blood pressure (SBP), mmHg** | **124,7 (18,5)** | **124,1 (18,3)** | **125,5 (18,7)** |
| **Fasting blood glucose, mg/dl** | **84,3 (28,2)** | **81,0 (24,3)** | **89,9 (33,1)** |
| **Blood total cholesterol, mg/dl** | **142,9 (45,7)** | **136,2 (36,5)** | **154,8 (56,5)** |
| ^1^ n (unweighted) (%); Mean (SD) | | | |

## Supplementary Table 7. Factors associated with overweight, and obese status as compared to normal weight further adjusted for rural/urban status (N=111,577).

| **Predictors** | **Underweight vs Normal** | |  | **Overweight vs Normal** | |  | **Obesity vs Normal** | |
| --- | --- | --- | --- | --- | --- | --- | --- | --- |
|  | **OR** | **95% CI** |  | **OR** | **95% CI** |  | **OR** | **95% CI** |
| **Sex** |  |  |  |  |  |  |  |  |
| Male | 1(ref) |  |  | 1(ref) |  |  | 1(ref) |  |
| Female | 1.25 | 1·06 — 1.46 |  | 2.05 | 1.81 — 2.33 |  | 4.86 | 4.08 — 5.84 |
| **Age groups** |  |  |  |  |  |  |  |  |
| [18;25) | 1(ref) |  |  | 1(ref) |  |  | 1(ref) |  |
| [25;35) | 0.84 | 0.66 — 1.07 |  | 1.86 | 1.50 — 2.32 |  | 2.86 | 2.01 — 4.15 |
| [35;45) | 0.87 | 0.67 — 1.14 |  | 2.46 | 1.97 — 3.1 |  | 5.5 | 3.85 — 8.03 |
| [45;55) | 1.00 | 0.76 — 1.32 |  | 2.55 | 2.00 — 3.24 |  | 6.68 | 4.64 — 9.89 |
| [55;69] | 1.25 | 0.94 — 1.66 |  | 2.63 | 2.04 — 3.38 |  | 6.71 | 4.62 — 9.99 |
| **Residency** |  |  |  |  |  |  |  |  |
| Rural | 1(ref) |  |  | 1(ref) |  |  | 1(ref) |  |
| Urban | 0.85 | 0.69 — 1.03 |  | 1.53 | 1.33 — 1.76 |  | 2.0 | 1.66 — 2.41 |
| **Level of education** |  |  |  |  |  |  |  |  |
| No education | 1(ref) |  |  | 1(ref) |  |  | 1(ref) |  |
| Primary education | 0.76 | 0.65 — 0.90 |  | 1.17 | 1.02 — 1.35 |  | 1.72 | 1.42 — 2.07 |
| Secondary education | 0.9 | 0.72 — 1.12 |  | 1.6 | 1.35 — 1.9 |  | 2.6 | 2.08 — 3.24 |
| Tertiary education | 0.96 | 0.66 — 1.37 |  | 1.9 | 1.49 — 2.41 |  | 3.32 | 2.45 — 4.50 |
| **Marital Status** |  |  |  |  |  |  |  |  |
| Single | 1(ref) |  |  | 1(ref) |  |  | 1(ref) |  |
| Married/Cohabitating | 0.82 | 0.68 — 1.01 |  | 1.1 | 0.93 — 1.3 |  | 1.02 | 0.83 — 1.24 |
| Divorced/Separated/Widowed | 1.11 | 0.85 — 1.47 |  | 0.87 | 0.7 — 1.09 |  | 0.72 | 0.55 — 0.94 |
| **Employment status** |  |  |  |  |  |  |  |  |
| Employee |  |  |  |  |  |  |  |  |
| Self-employed | 1.48 | 1.12 — 1.97 |  | 0.88 | 0.73 — 1.05 |  | 0.82 | 0.65 — 1.03 |
| Student voluntary | 1.79 | 1.31 — 2.47 |  | 0.62 | 0.48 — 0.8 |  | 0.55 | 0.39 — 0.78 |
| Unemployee / retreat | 1.53 | 1.15 — 2.04 |  | 0.78 | 0.65 — 0.94 |  | 0.75 | 0.59 — 0.94 |
| **Physical activity** |  |  |  |  |  |  |  |  |
| ≥150 min/wk moderate or ≥75 min/wk vigorous or ≥150 combination | 1(ref) |  |  | 1(ref) |  |  | 1(ref) |  |
| 1–149 min/wk moderate or 1–74 min/wk vigorous or 1–149 min/wk combination | 1.2 | 0.97 — 1.48 |  | 1.06 | 0.91 — 1.24 |  | 1.27 | 1.04 — 1.54 |
| None | 1.2 | 0.99 — 1.46 |  | 1.05 | 0.91 — 1.22 |  | 1.27 | 1.05 — 1.54 |
| **Fruit & vegetable consumption** |  |  |  |  |  |  |  |  |
| 4–5 servings per day | 1(ref) |  |  | 1(ref) |  |  | 1(ref) |  |
| 2–3 servings per day | 0.88 | 0.75 — 1.03 |  | 1.14 | 1.01 — 1.28 |  | 1.18 | 1.01 — 1.38 |
| 0–1 serving per day | 1.16 | 0.92 — 1.44 |  | 1 | 0.84 — 1.19 |  | 1.33 | 1.07 — 1.64 |
| **Smoking** |  |  |  |  |  |  |  |  |
| Never or quit > 12 m | 1(ref) |  |  | 1(ref) |  |  | 1(ref) |  |
| Former. quit ≤12 m | 1.53 | 0.62 — 3.41 |  | 0.13 | 0.02 — 0.54 |  | 0.89 | 0.29 — 2.39 |
| Current | 1.83 | 1.47 — 2.25 |  | 0.63 | 0.51 — 0.78 |  | 0.63 | 0.47 — 0.86 |
| **Alcohol consumption** |  |  |  |  |  |  |  |  |
| Never or quit > 12 m | 1(ref) |  |  | 1(ref) |  |  | 1(ref) |  |
| Current drinker | 0.96 | 0.8 — 1.15 |  | 1.04 | 0.9 — 1.2 |  | 1 | 0.83 — 1.21 |
| Heavy episodic drink | 0.98 | 0.79 — 1.22 |  | 1.02 | 0.85 — 1.22 |  | 1.06 | 0.84 — 1.35 |
| OR: Odds Ratio, CI = Confidence Interval. Data are in OR (95% CI).  OR and their 95% CI were estimated by hierarchical multinomial mixed-effects regression using normal weight as the reference category and including a random effect on the country and year; they were adjusted for the variables listed in the Table. The analysis is conducted in the subsample with available rural-status data and comprising 111577 participants. | | | | | | | | |

## Supplementary Table 8. Population Attributable Risk of high blood pressure due to abnormal BMI Categories

|  | **Study outcome** | | | | | | | | |
| --- | --- | --- | --- | --- | --- | --- | --- | --- | --- |
| **Exposure** | **High-normal BP** | | | **Grade 1 Hypertension** | | | **Grade 2 Hypertension** | | |
|  | **Pe¹** | **OR [CI]** | **PAF (%)** | **Pe¹** | **OR [CI]** | **PAF (%)** | **Pe¹** | **OR [CI]** | **PAF (%)** |
| **BMI Category** |  |  |  |  |  |  |  |  |  |
| Underweight | 9.4% | 0.95 [0.88-1.02] | -0.47 | 7.4% | 1.06 [0.97-1.15] | 0.44 | 7.4% | 1.76 [1.55-2.01] | 5.32 |
| Overweight | 21.4% | 1.40 [1.34-1.47] | 7.97 | 22.7% | 1.60 [1.52-1.68] | 11.99 | 24.9% | 1.97 [1.82-2.12] | 19.45 |
| Obesity | 11.0% | 1.83 [1.73-1.94] | 8.37 | 13.8% | 2.65 [2.48-2.83] | 18.55 | 19.0% | 4.10 [3.73-4.48] | 37.07 |
| 1.Pe = prevalence of exposure in study population  2.PAF = Population attributable fraction calculated as Pe * (OR-1)/(Pe *(OR-1)+1) .  OR: Odds ratios, CI = Confidence Interval, BP = Blood Pressure  OR and their 95% CI were estimated using a hierarchical multinomial mixed-effects regression, with normal BP as the reference outcome category and normal weight as the reference exposure category, and including random effects for country and year. The estimates were adjusted for individual covariates such as sex, age, education level, smoking, alcohol consumption, physical activity, and fruit and vegetable consumption. | | | | | | | | | |

## Supplementary Table 9. Population Attributable Risk of High Blood Sugar due to abnormal BMI Categories

|  | **Study outcome** | | | | | |
| --- | --- | --- | --- | --- | --- | --- |
| **Exposure** | **Prediabetes** | | | **Diabetes** | | |
|  | **Pe¹** | **OR [CI]** | **PAF (%)** | **Pe¹** | **OR[CI]** | **PAF (%)** |
| **BMI Category** |  |  |  |  |  |  |
| Underweight | 9.5% | 1.03 [0.95-1.11] | 0.28 | 7.8% | 1.03 [0.89-1.18] | 0.23 |
| Overweight | 22.3% | 1.23 [1.16-1.31] | 4.88 | 28.0% | 1.55 [1.42-1.69] | 13.33 |
| Obesity | 16.4% | 1.65 [1.55-1.76] | 9.46 | 22.9% | 2.53 [2.32-2.78] | 26.11 |
| 1.Pe = prevalence of exposure in study population  2.PAF = Population attributable fraction calculated as Pe * (OR-1)/(Pe *(OR-1)+1) .  OR: Odds ratios, CI = Confidence Interval.  OR and their 95% CI were estimated using a hierarchical multinomial mixed-effects regression, with nondiabetic as the reference outcome category and normal weight as the reference exposure category, and including random effects for country and year. The estimates were adjusted for individual covariates such as sex, age, education level, smoking, alcohol consumption, physical activity, and fruit and vegetable consumption. | | | | | | |

# **Appendix 5: Figures**

**N = 215499**

N = **203608**

***Reason for exclusion***

- Participants with missing data on Sex assigned at birth, **N=39**
- Participants with age less than 18 and above 69, **N=5341**
- Pregnant women, **N=65**11

**Analysis on BMI**

**N =198901**

***Reason for exclusion***

- Participants with missing data on BMI, **N=4**707

Overall selected participants

**N = 215724**

***Reason for exclusion***

- Invalid records, **N=225**

## Supplementary Figure 1: Flowchart of the study.


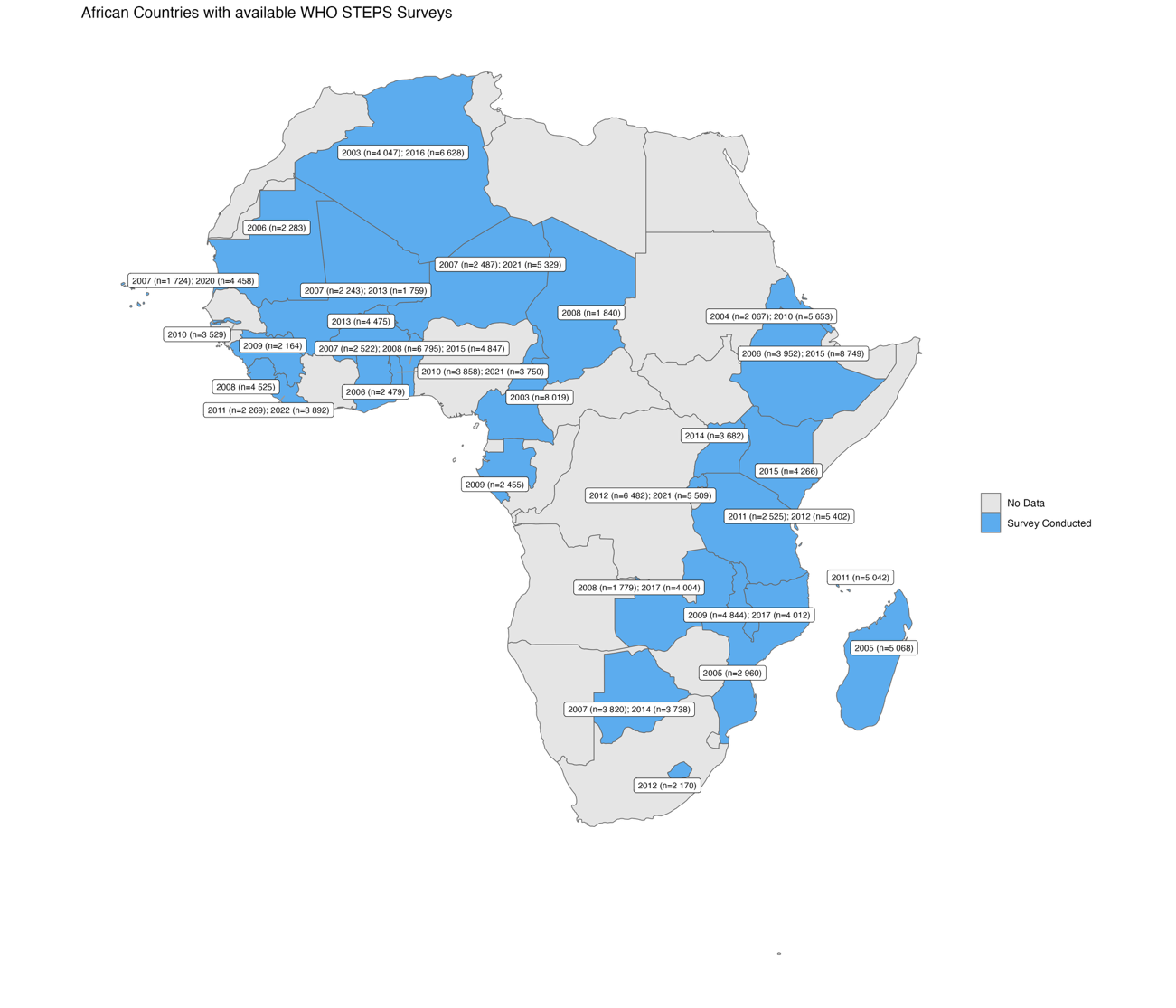


## Supplementary Figure 2. Map of African countries with available WHO STEPS Surveys included in the study (2003–2022)


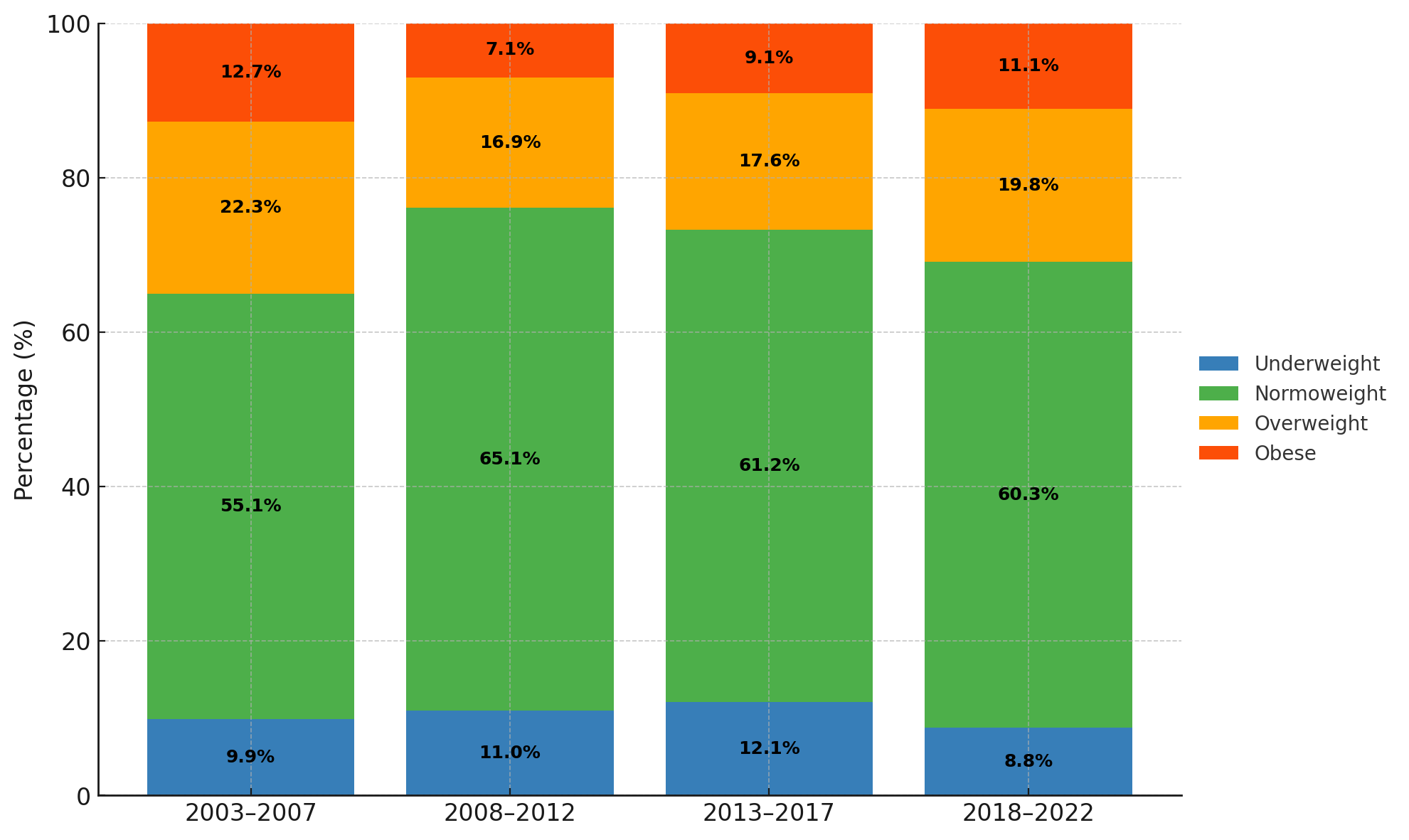


## Supplementary Figure 3. Distribution of BMI categories by study periods (2003–2022)

**
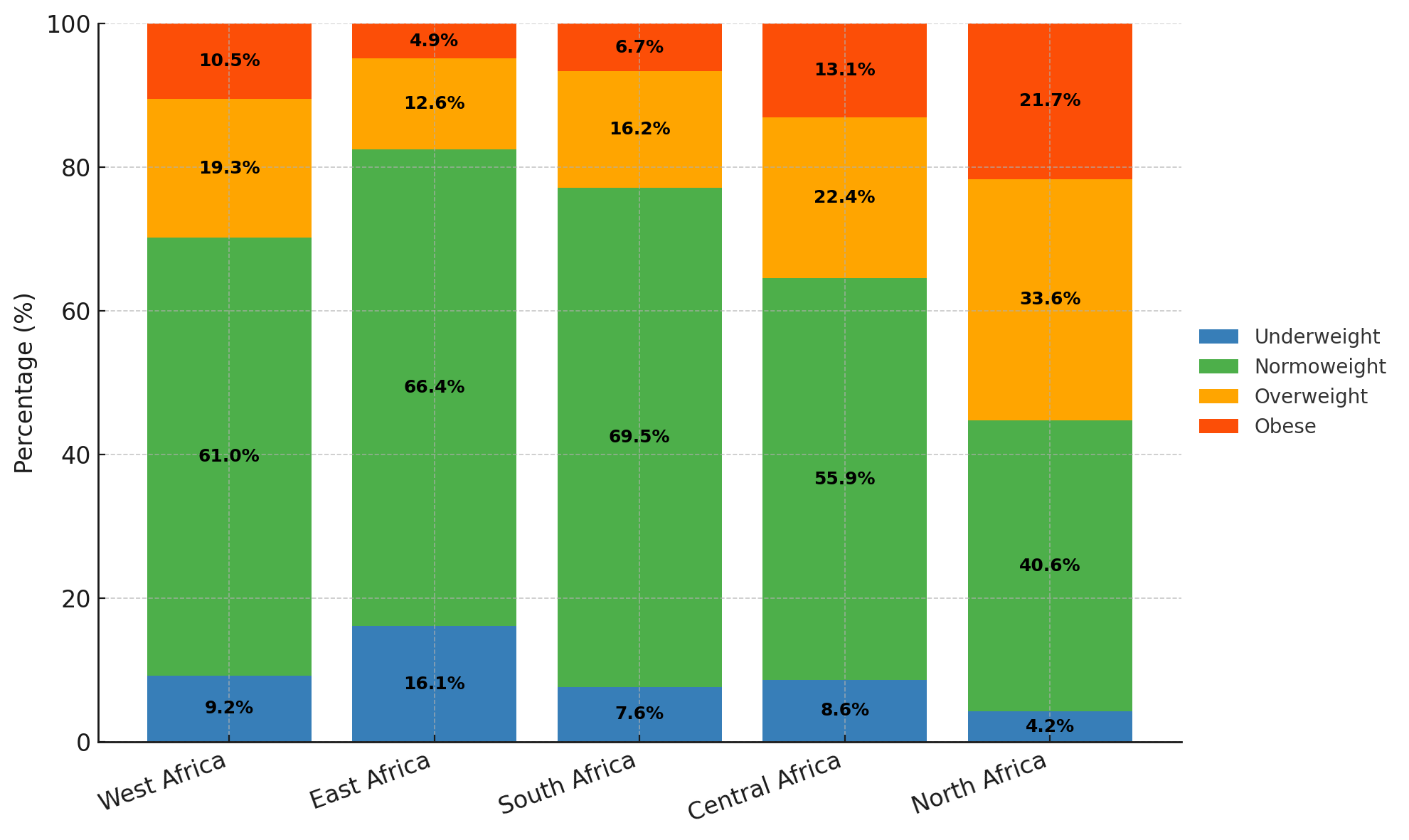
**

## Supplementary Figure 4. Distribution of BMI categories by sub regions (2003–2022)


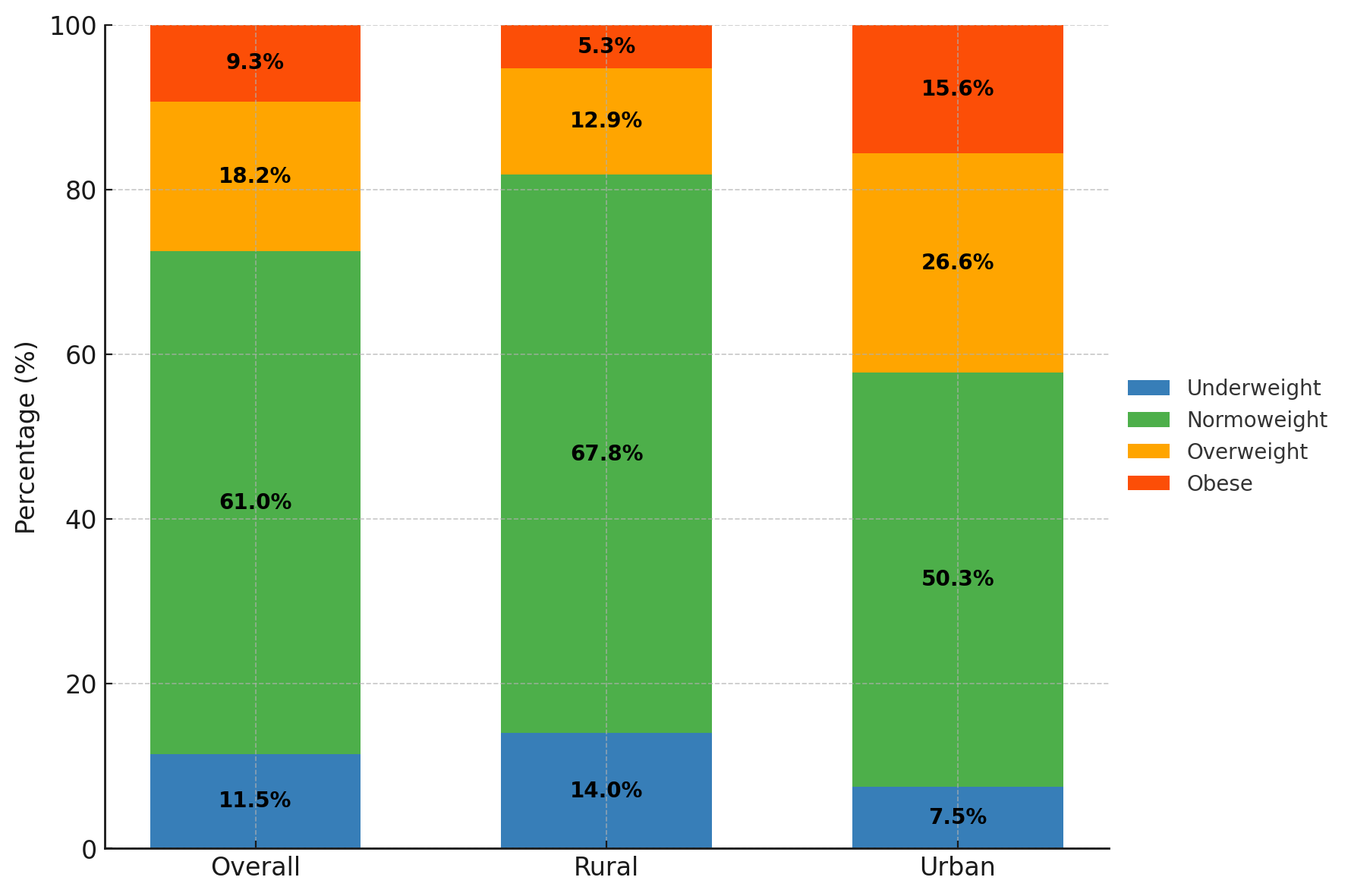


## Supplementary Figure 5. Distribution of BMI categories overall and by Urban and Rural Residence (N=111,577)

**
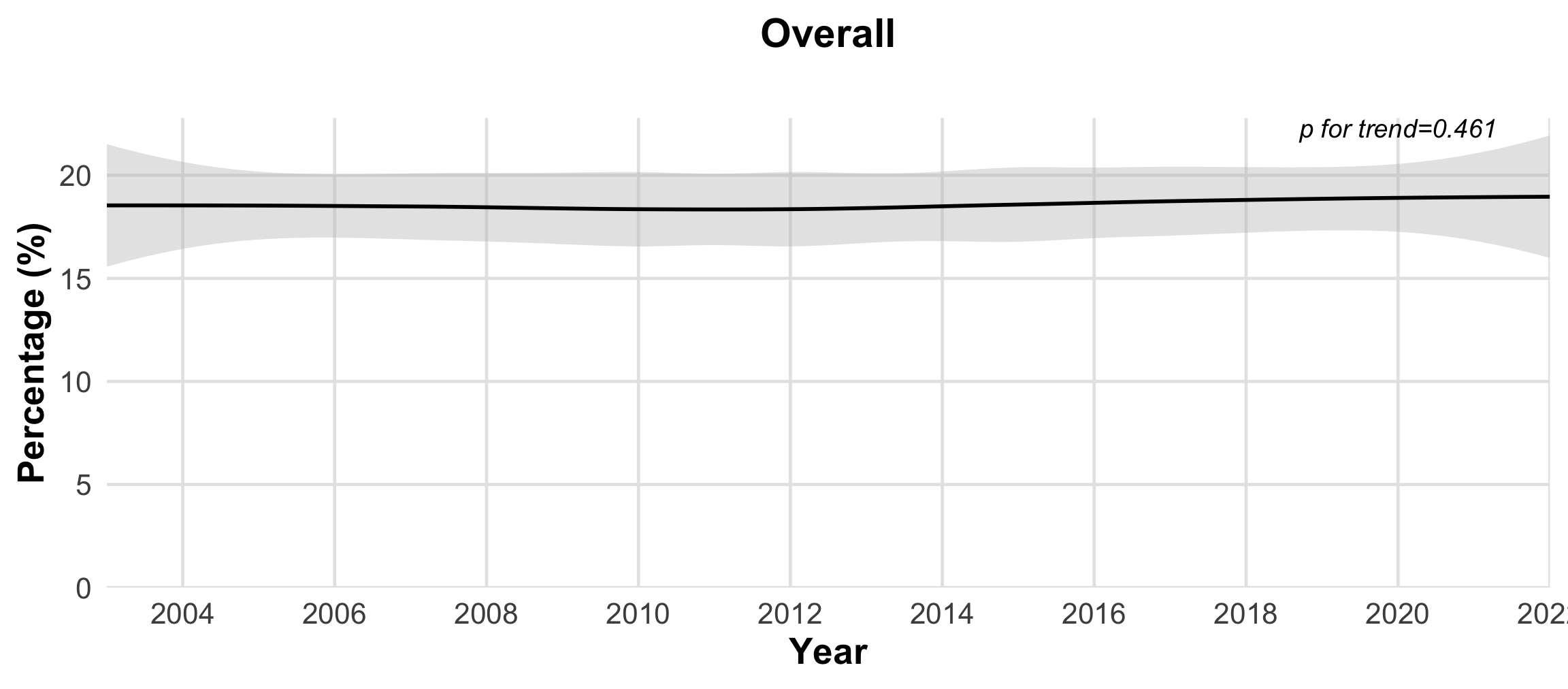
**

**
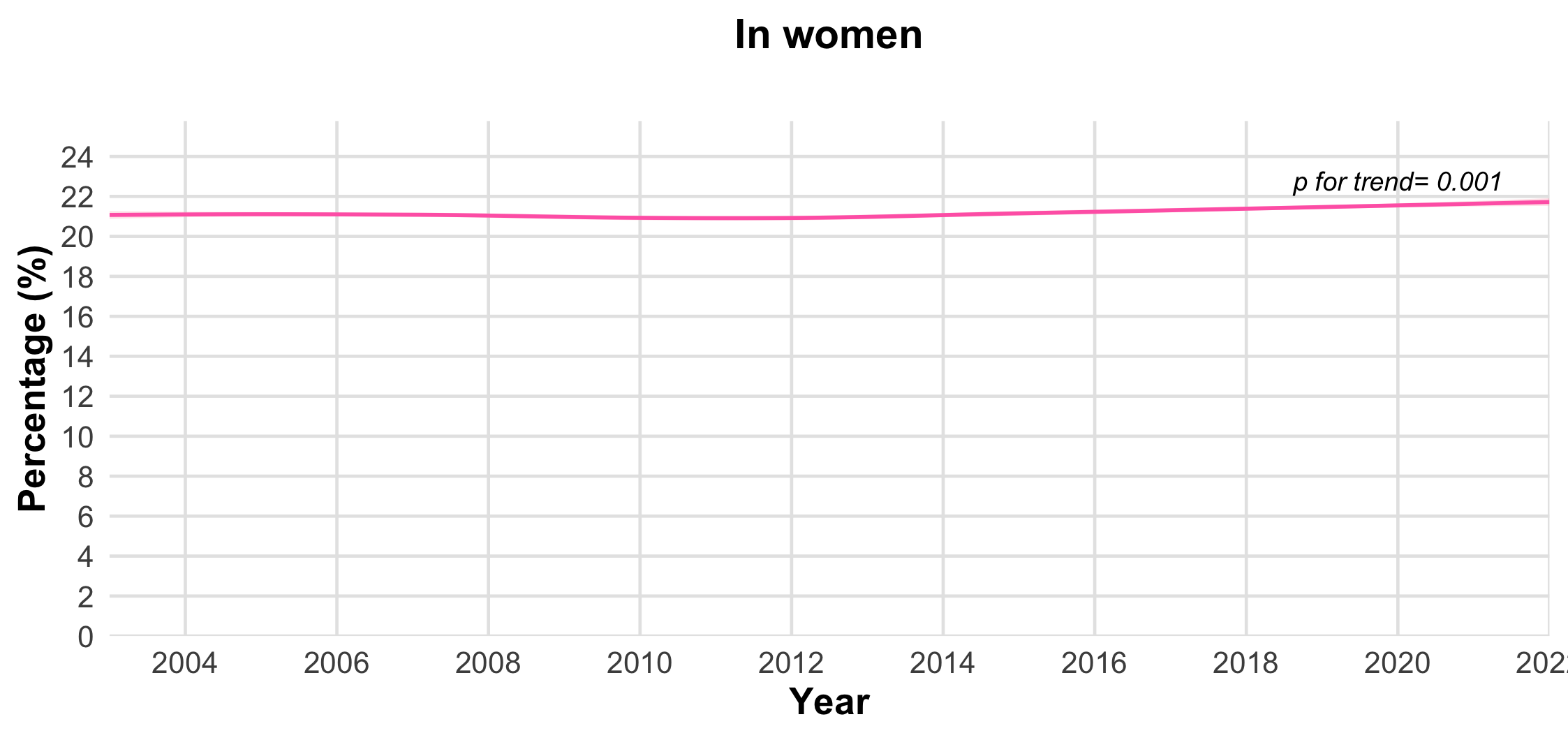
**

**
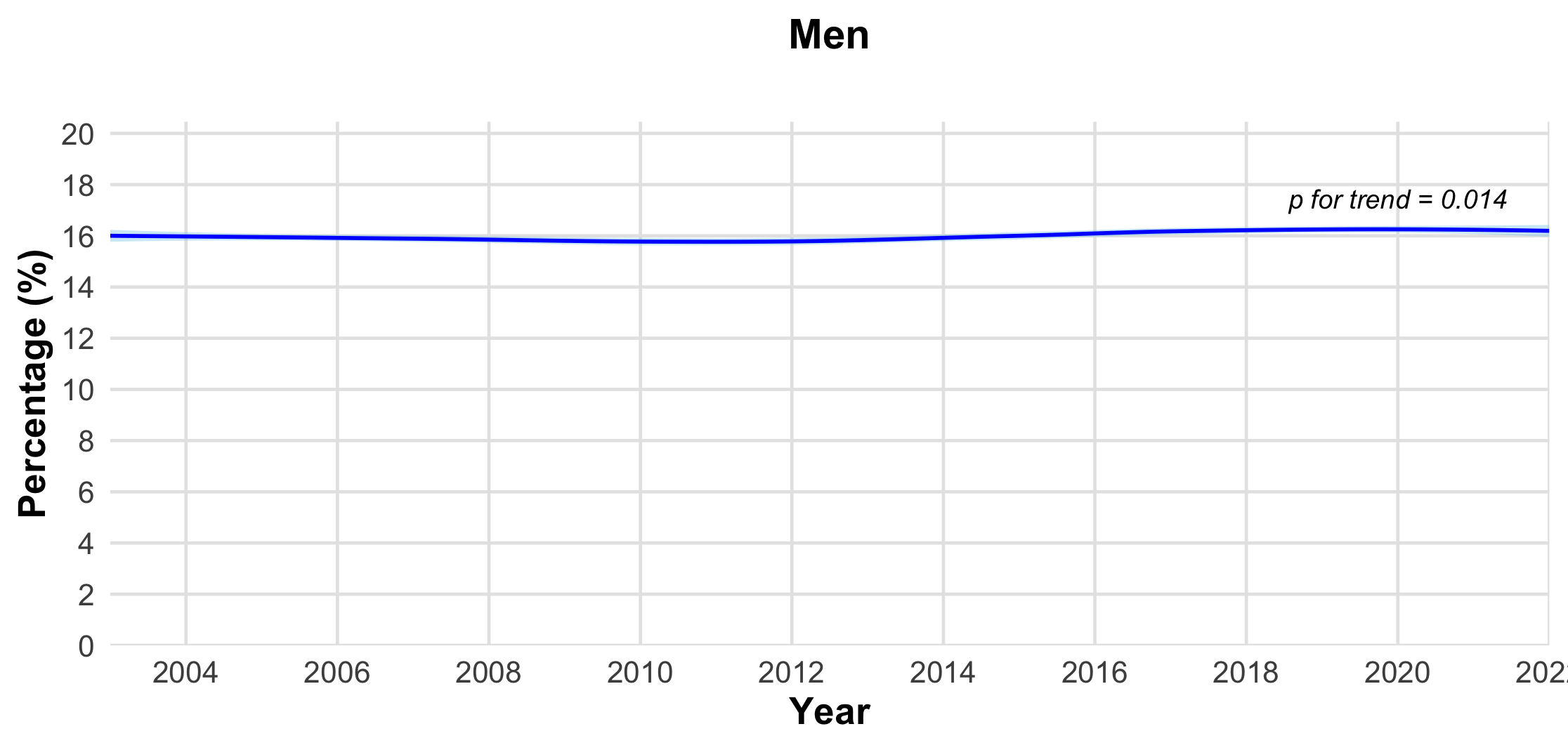
**

## Supplementary Figure 6. Trend in age-standardised prevalence of Overweight overall and by sex from 2003 – 2022. Imputed data for all the 47 countries in the WHO African region


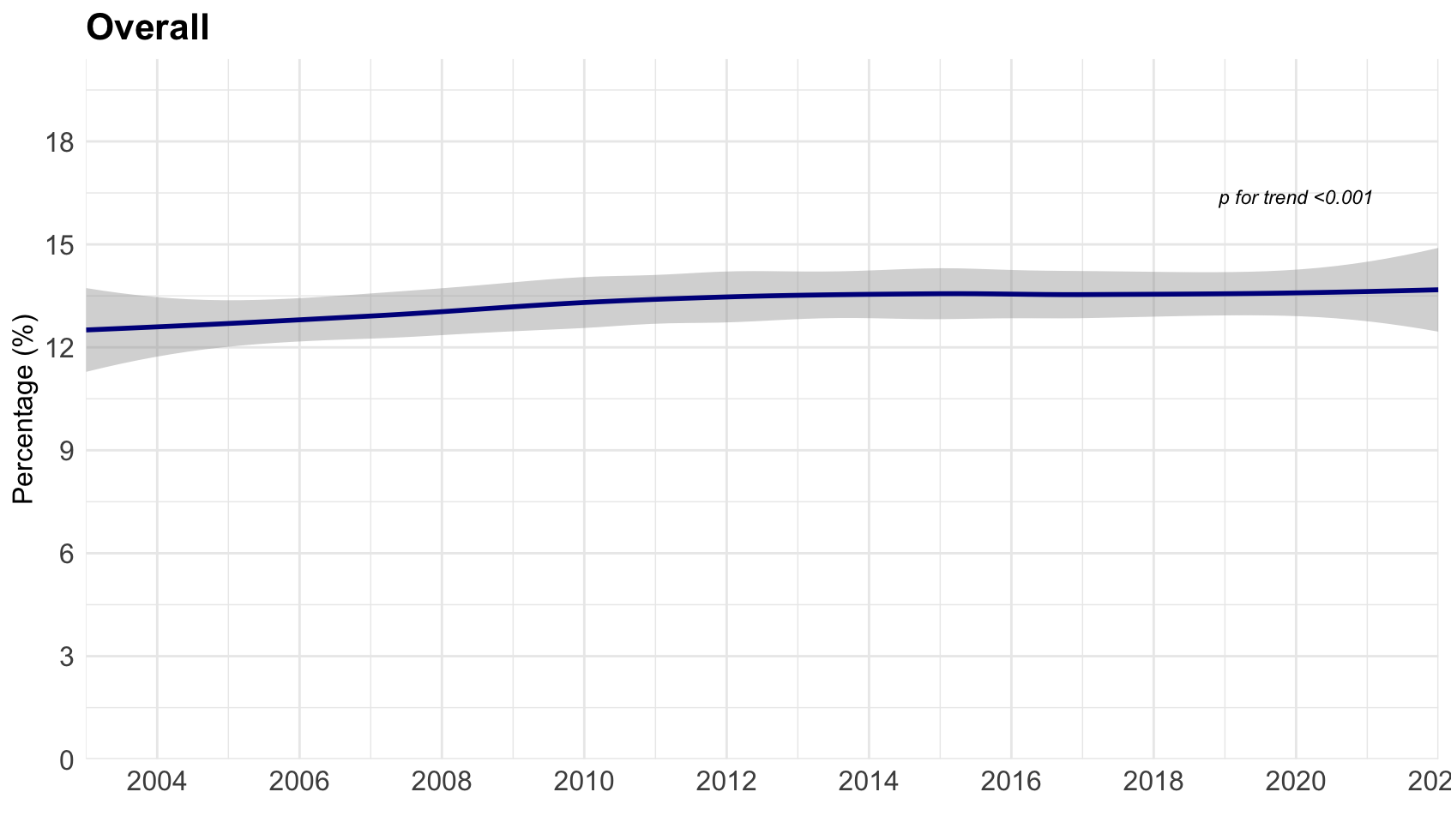


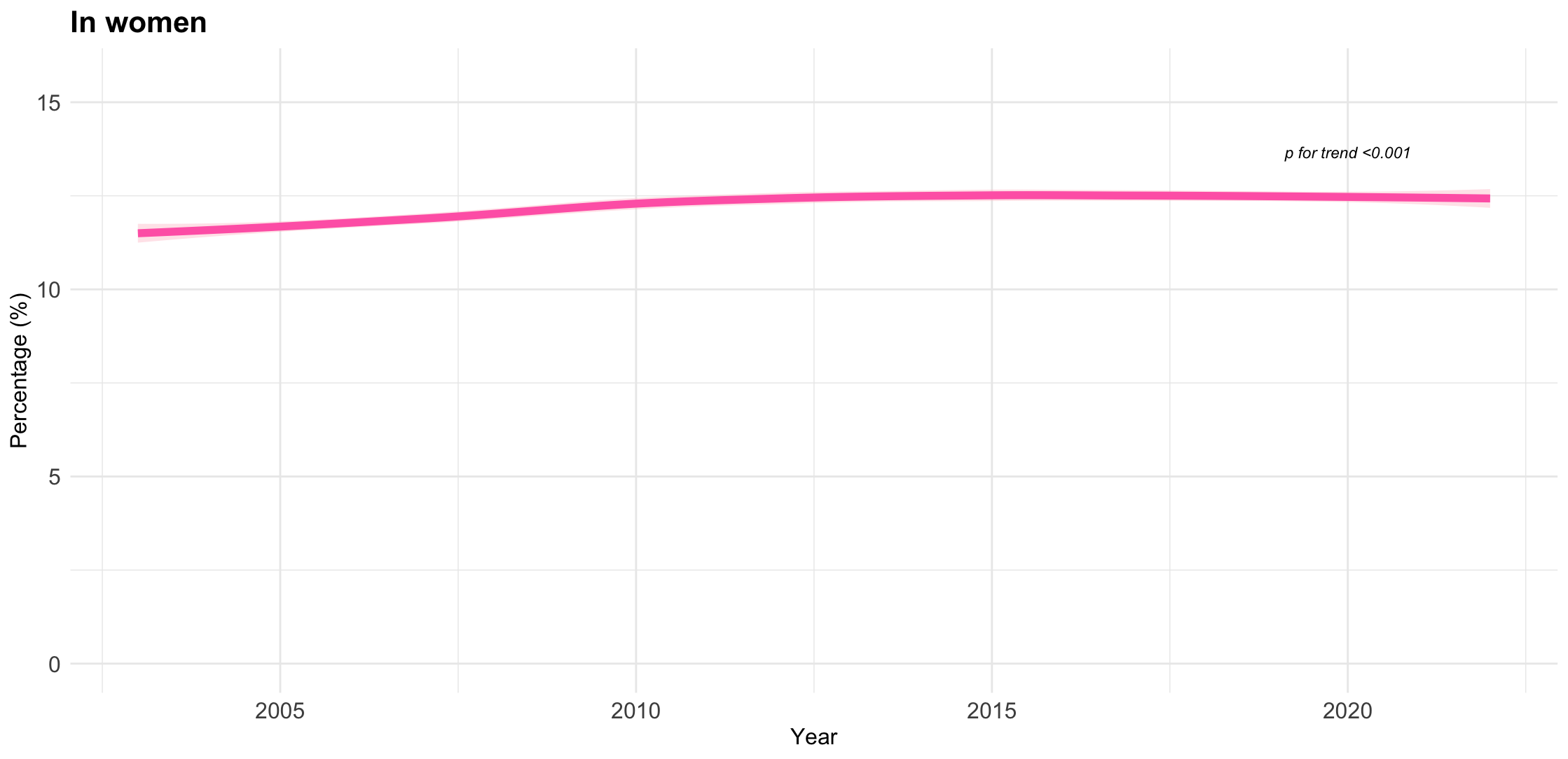


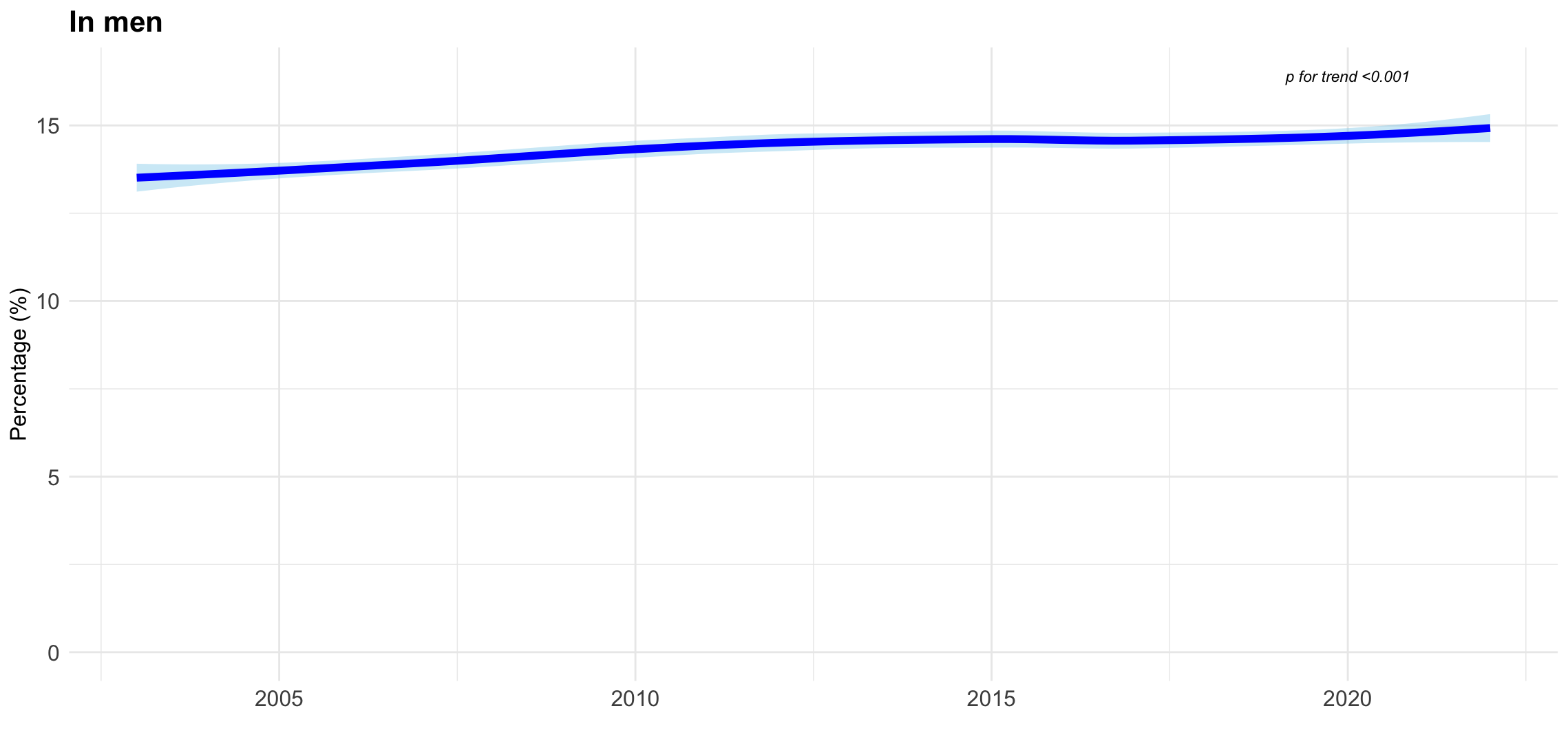


## Supplementary Figure 7. Trend in age-standardised prevalence of underweight, overall and by sex from 2003 – 2022. Imputed data for all the 47 countries in the WHO African region


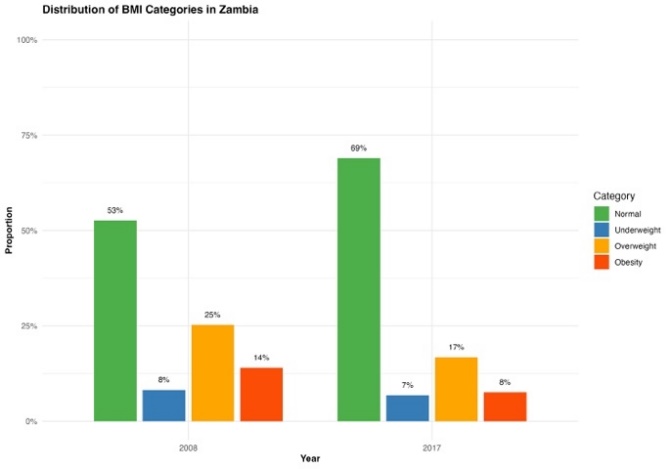

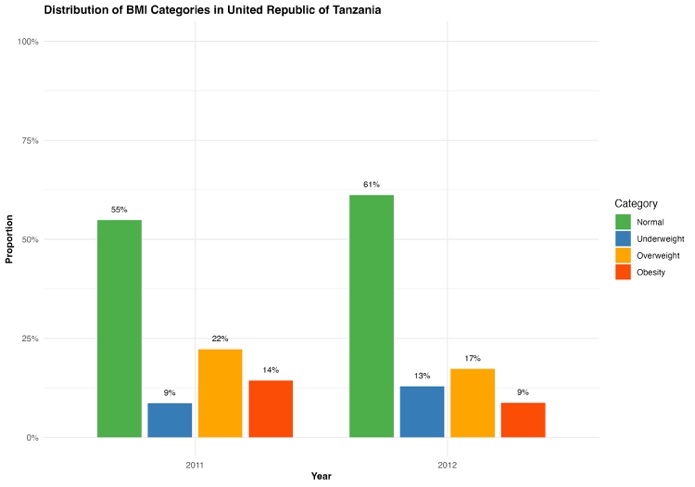

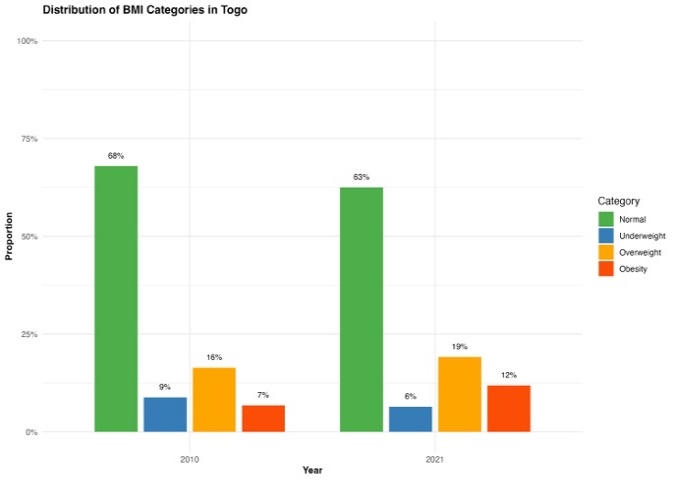

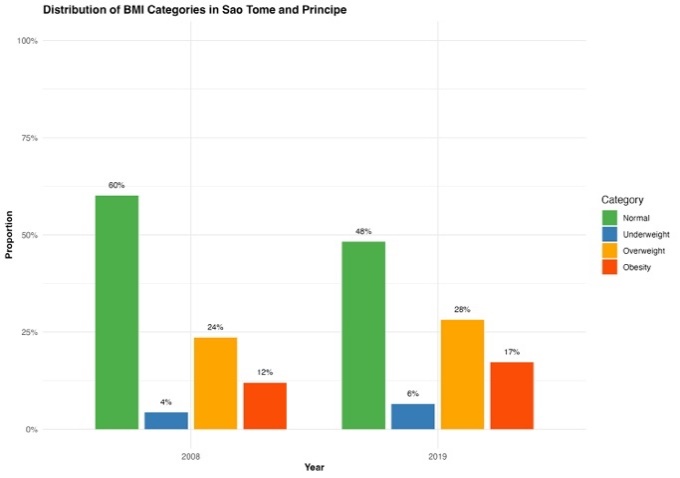

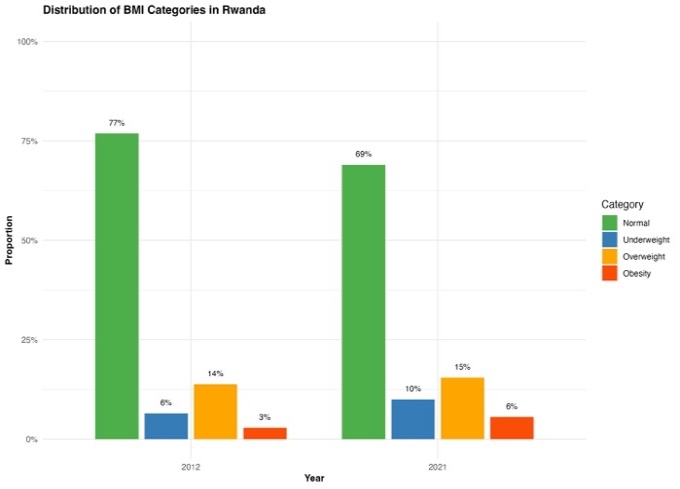

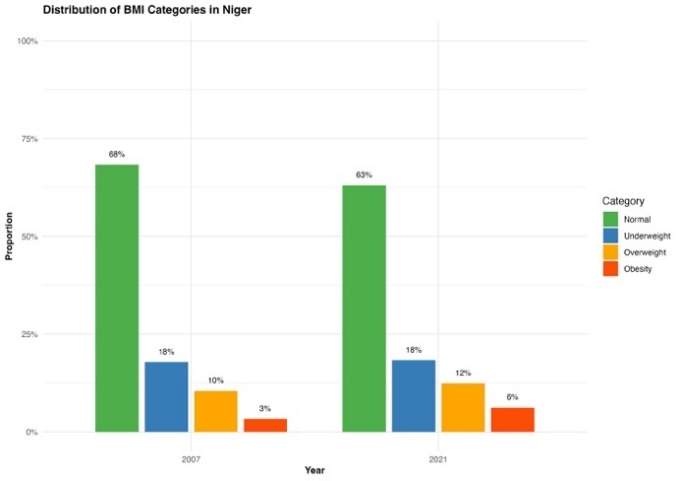


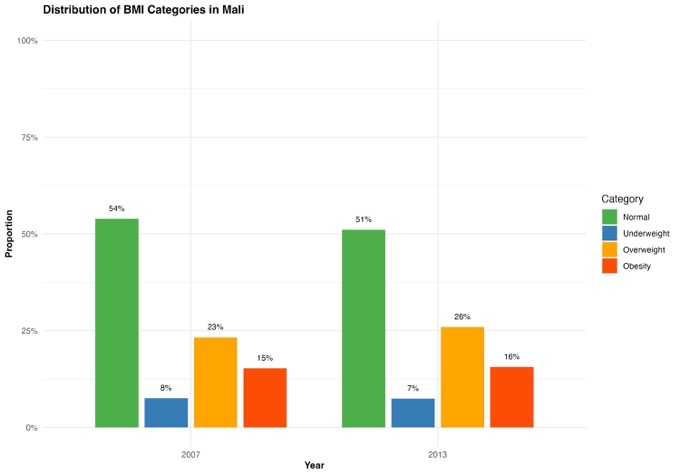

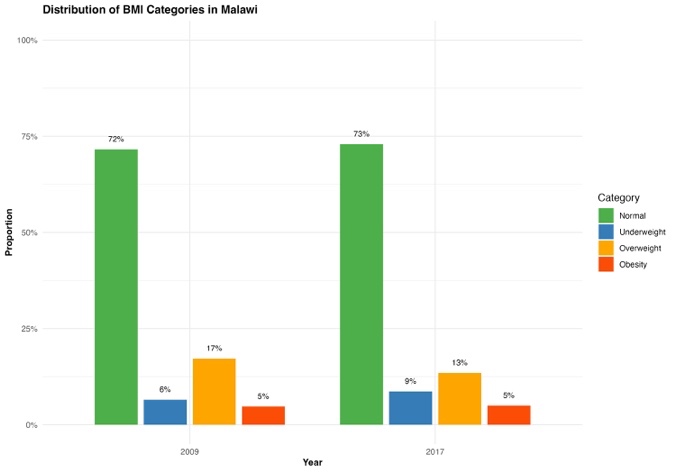

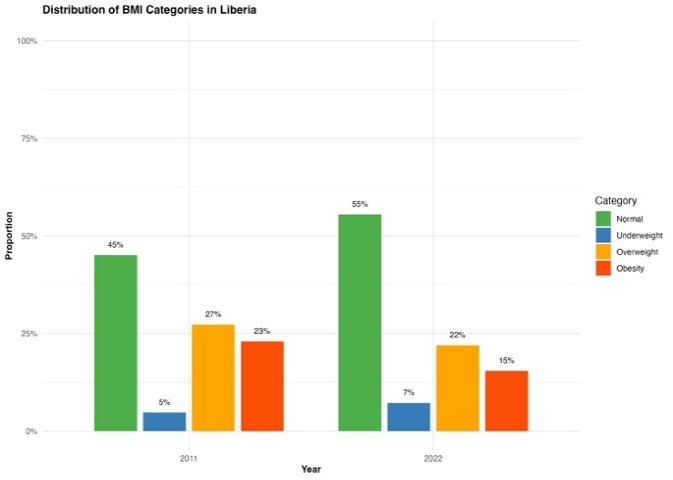

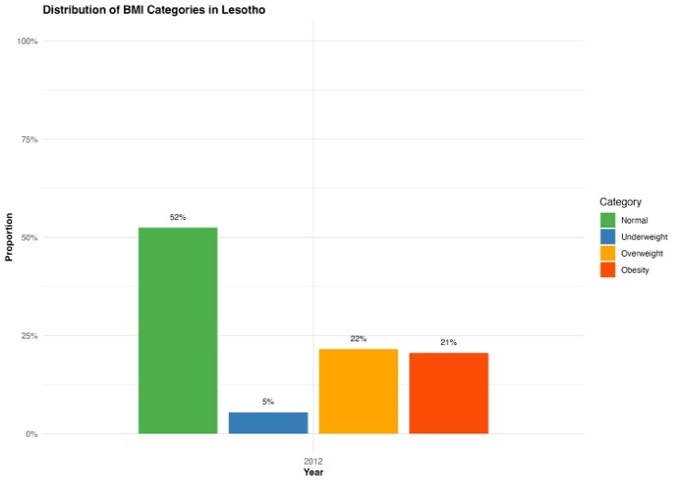

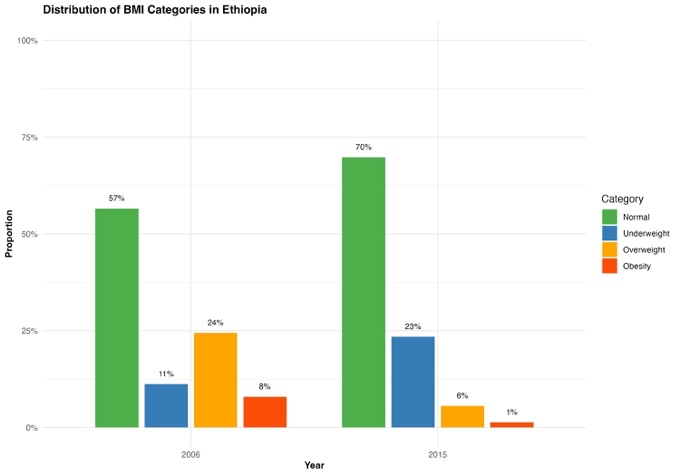

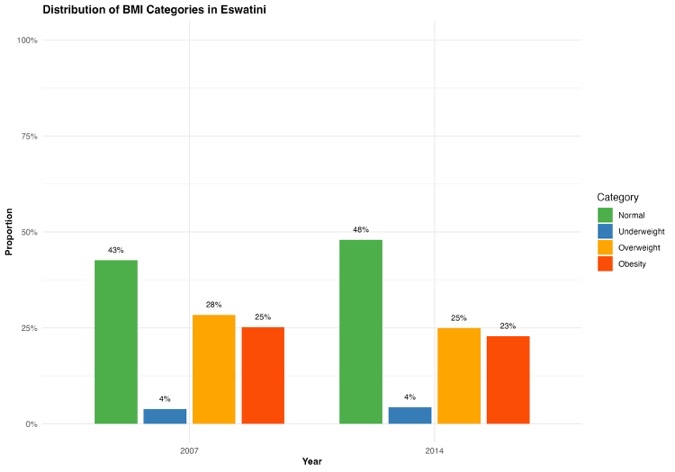


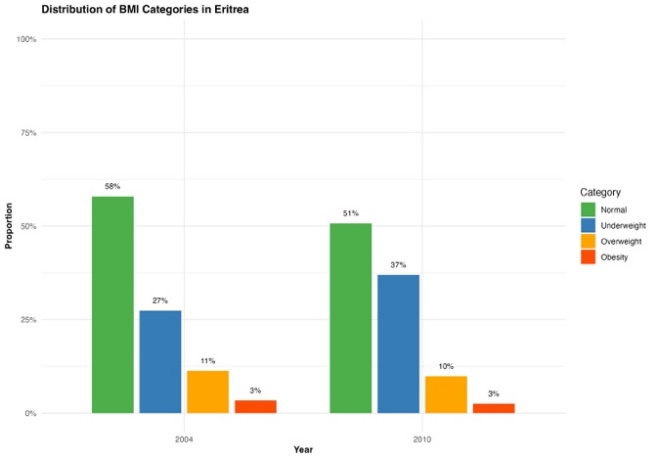

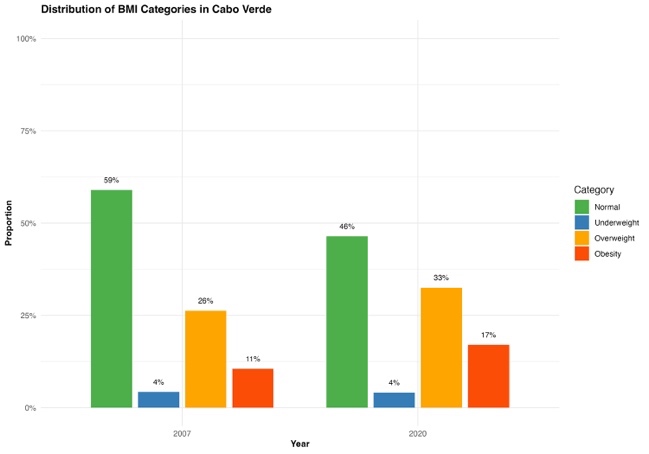

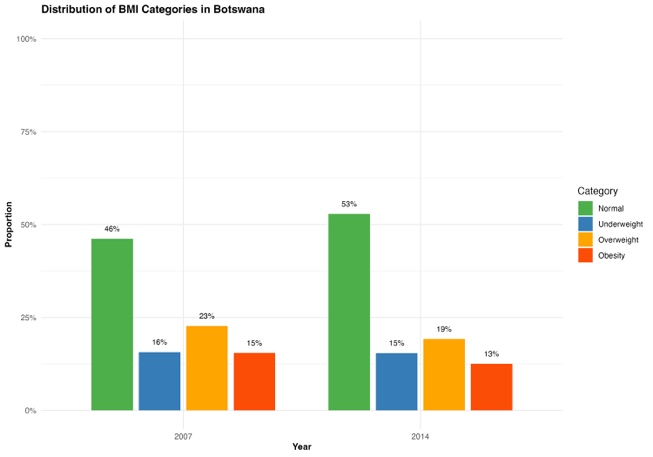

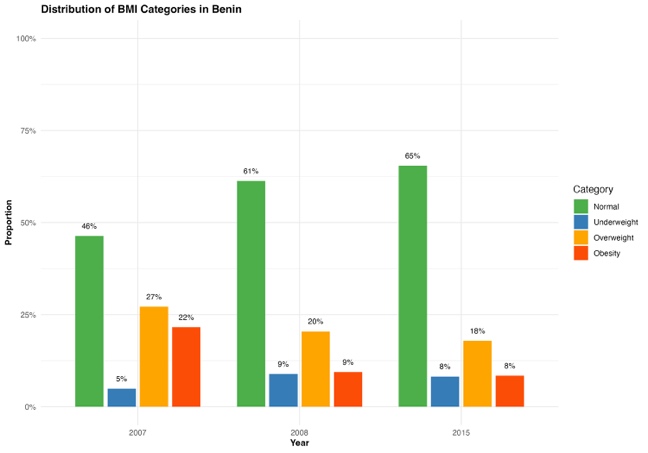

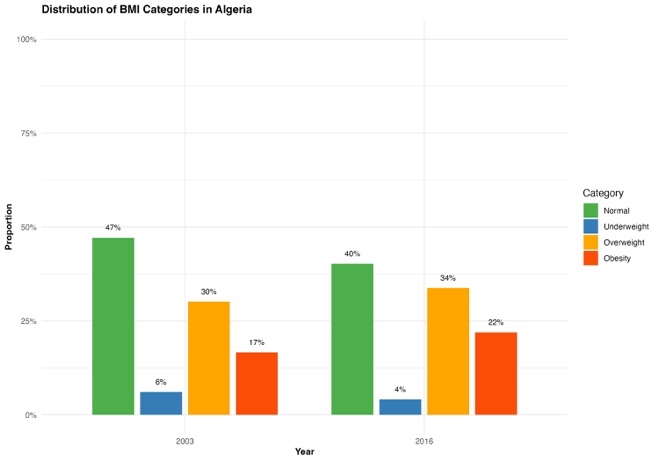


## Supplementary Figure 8. Distribution of BMI categories by country


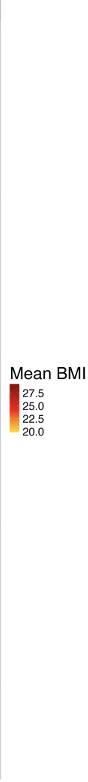

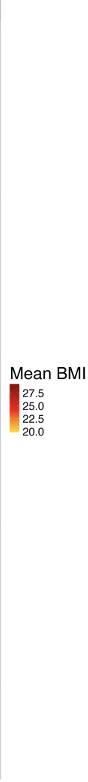

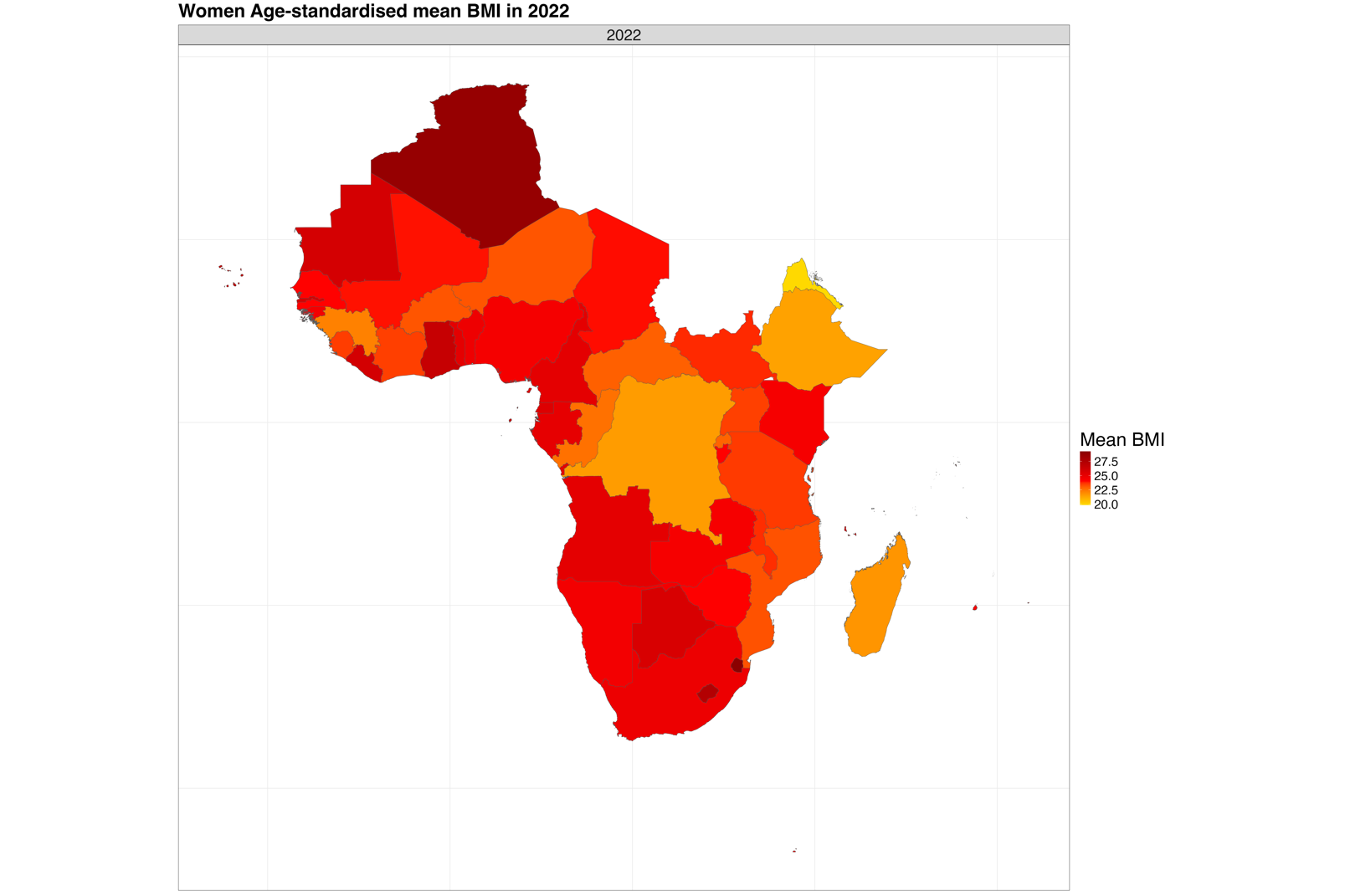

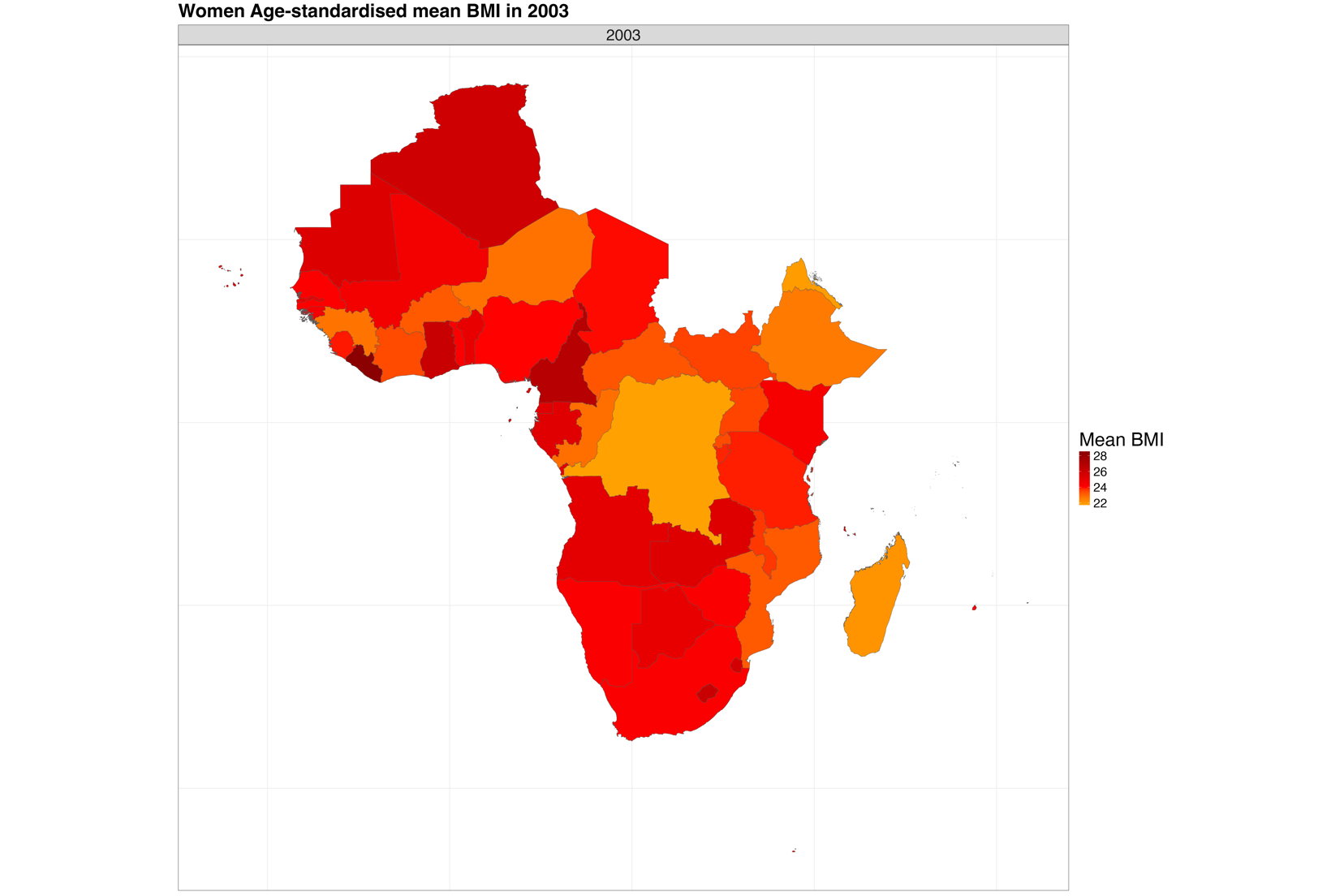

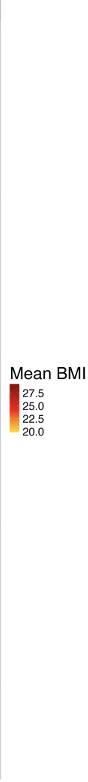

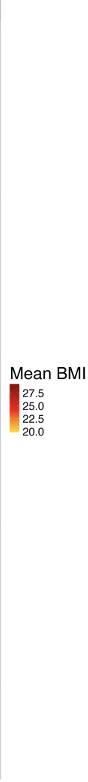


All estimates derive from the spatio-temporal model

## Supplementary Figure 9. Estimated age-standardised mean BMI in women by country in 2003 and 2022


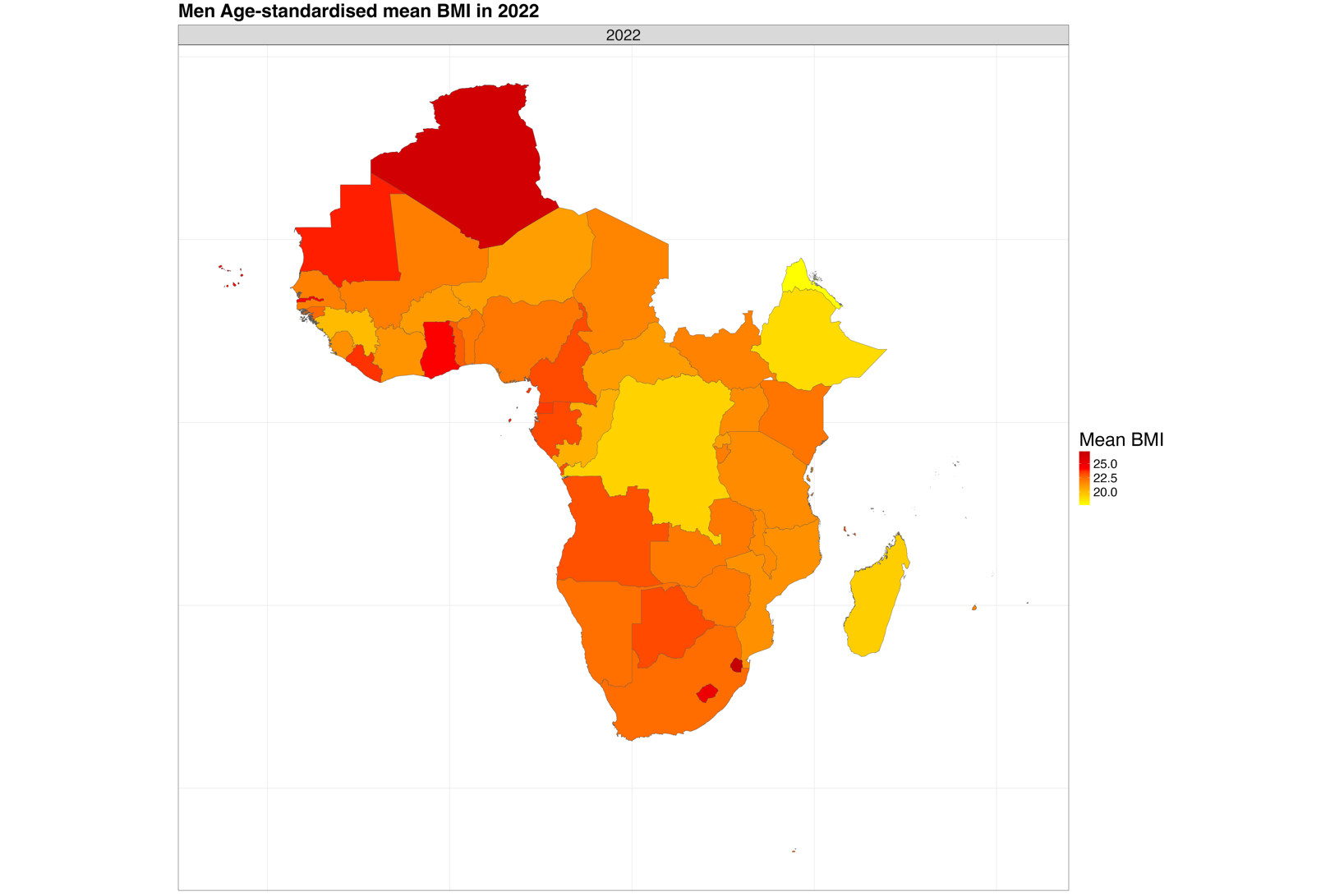

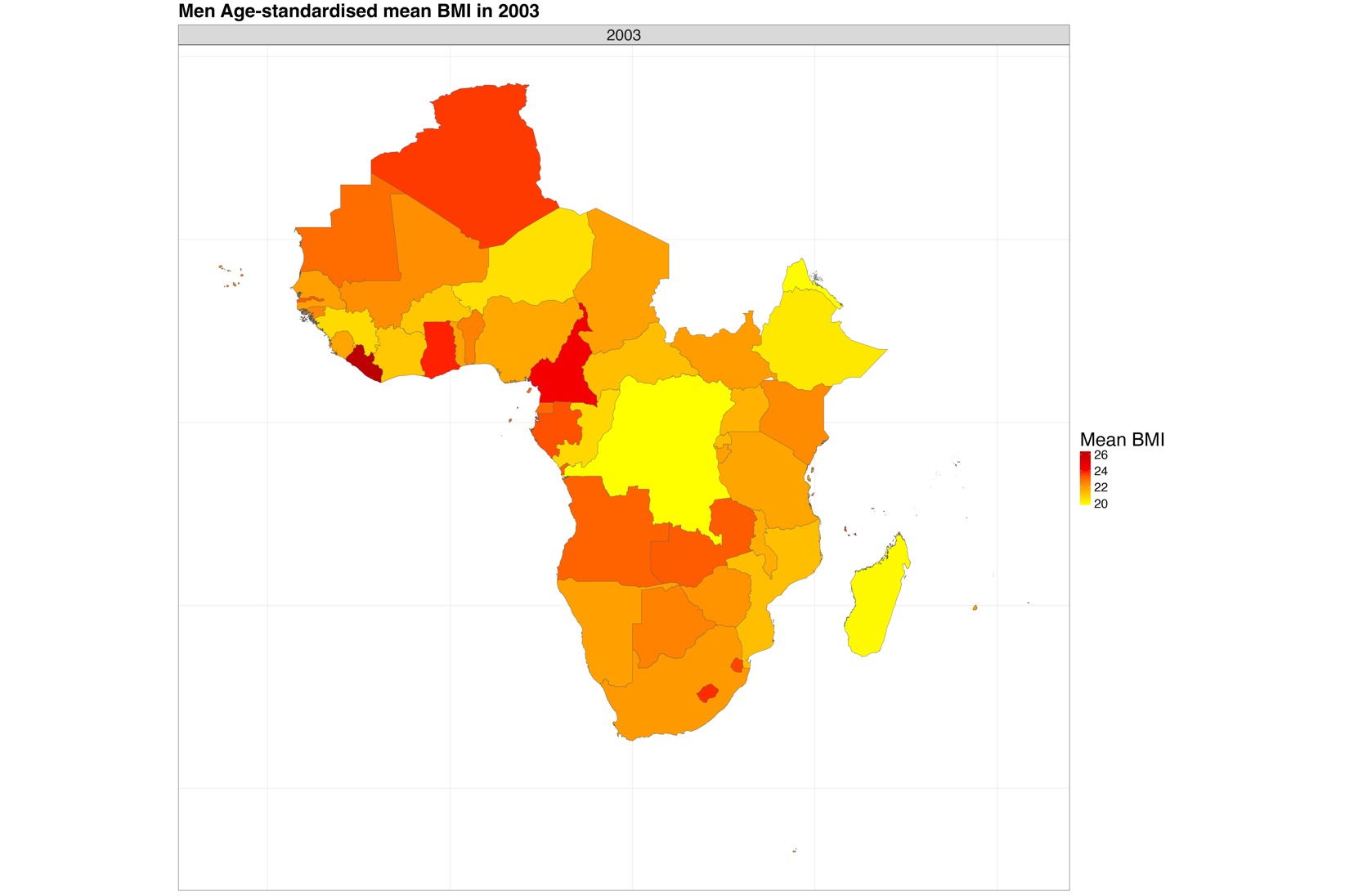

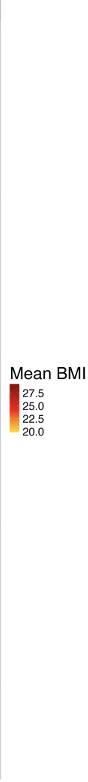

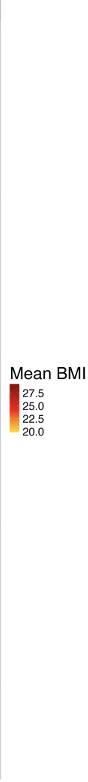


All estimates derive from the spatio-temporal model

## Supplementary Figure 10. Estimated age-standardised mean BMI in men by country in 2003 and 2022

**
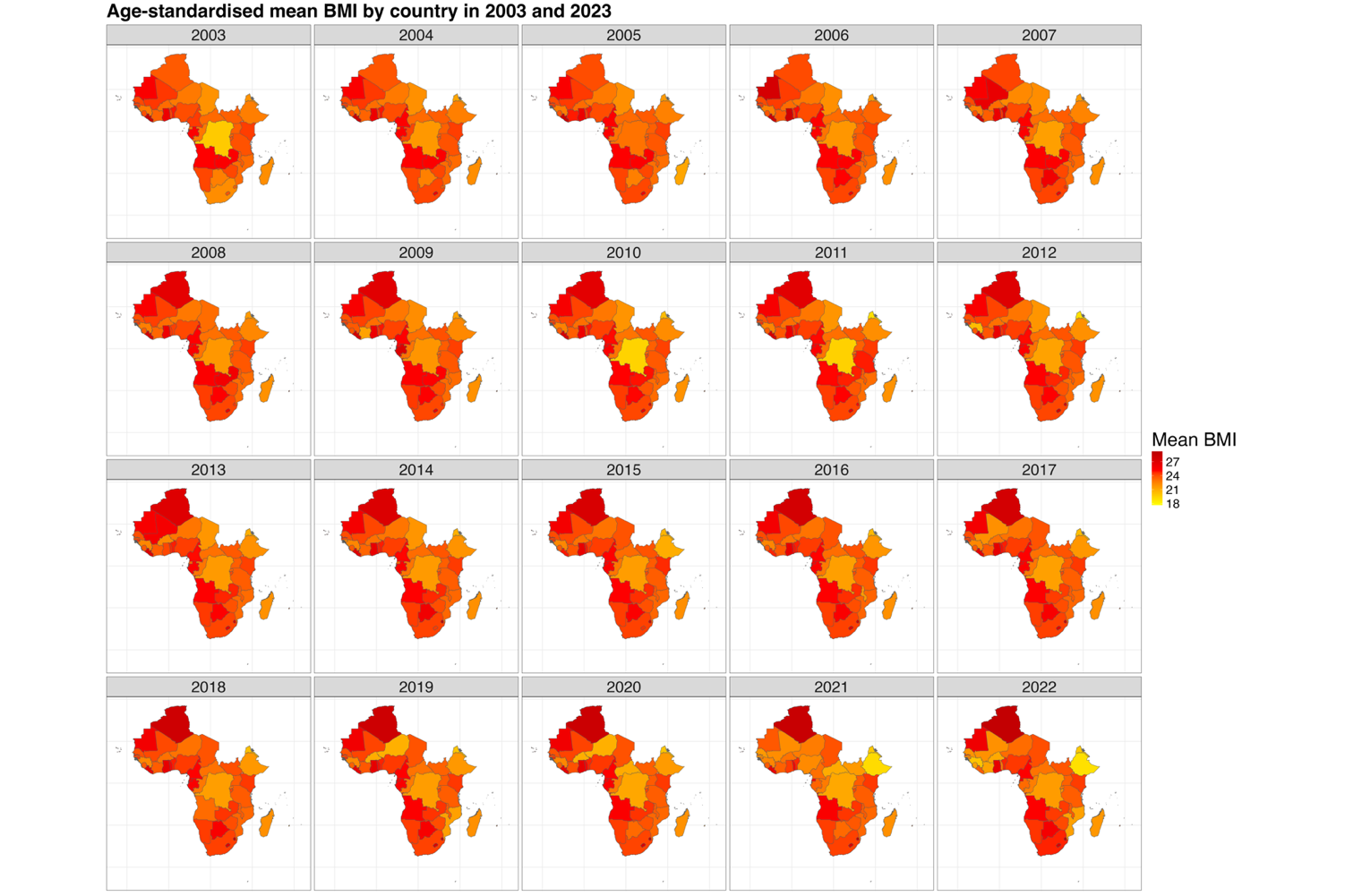
**

All estimates derive from the spatio-temporal model

## Supplementary Figure 11. Estimated age-standardized mean BMI by country and by year from 2003 to 2022


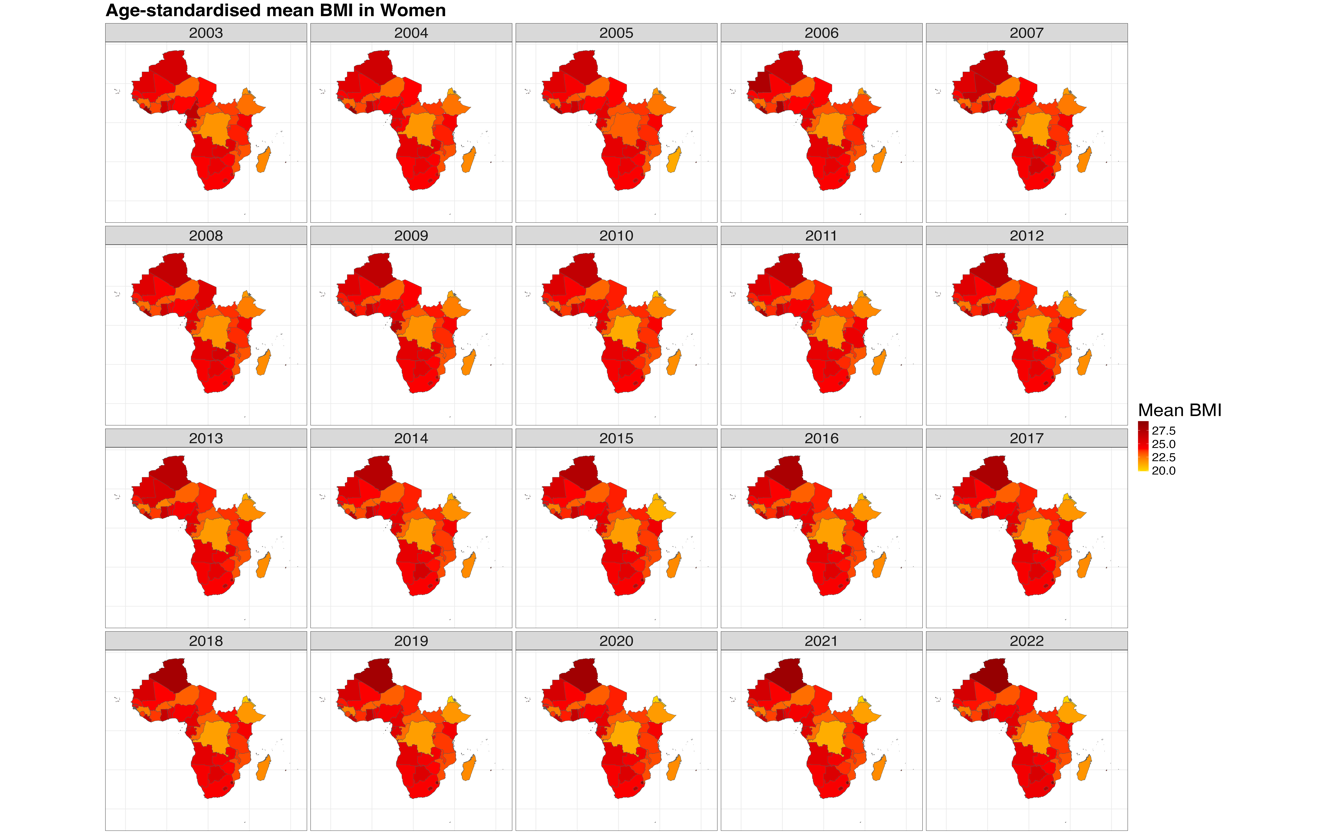


All estimates derive from the spatio-temporal model

## Supplementary Figure 12. Estimated age-standardized mean BMI in women by country and by year from 2003 to 2022


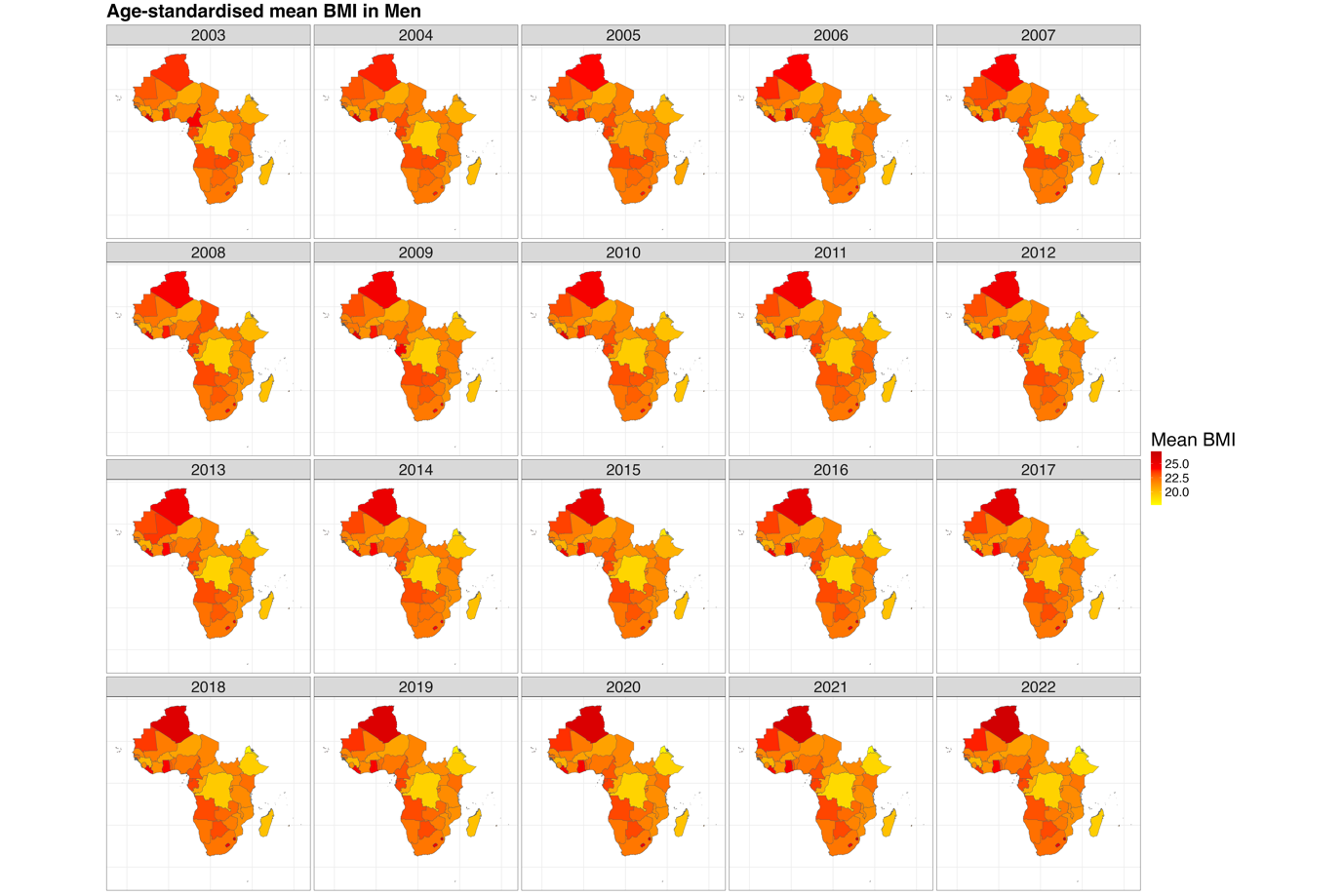


All estimates derive from the spatio-temporal model

## Supplementary Figure 13. Estimated age-standardized mean BMI in men by country and by year from 2003 to 2022


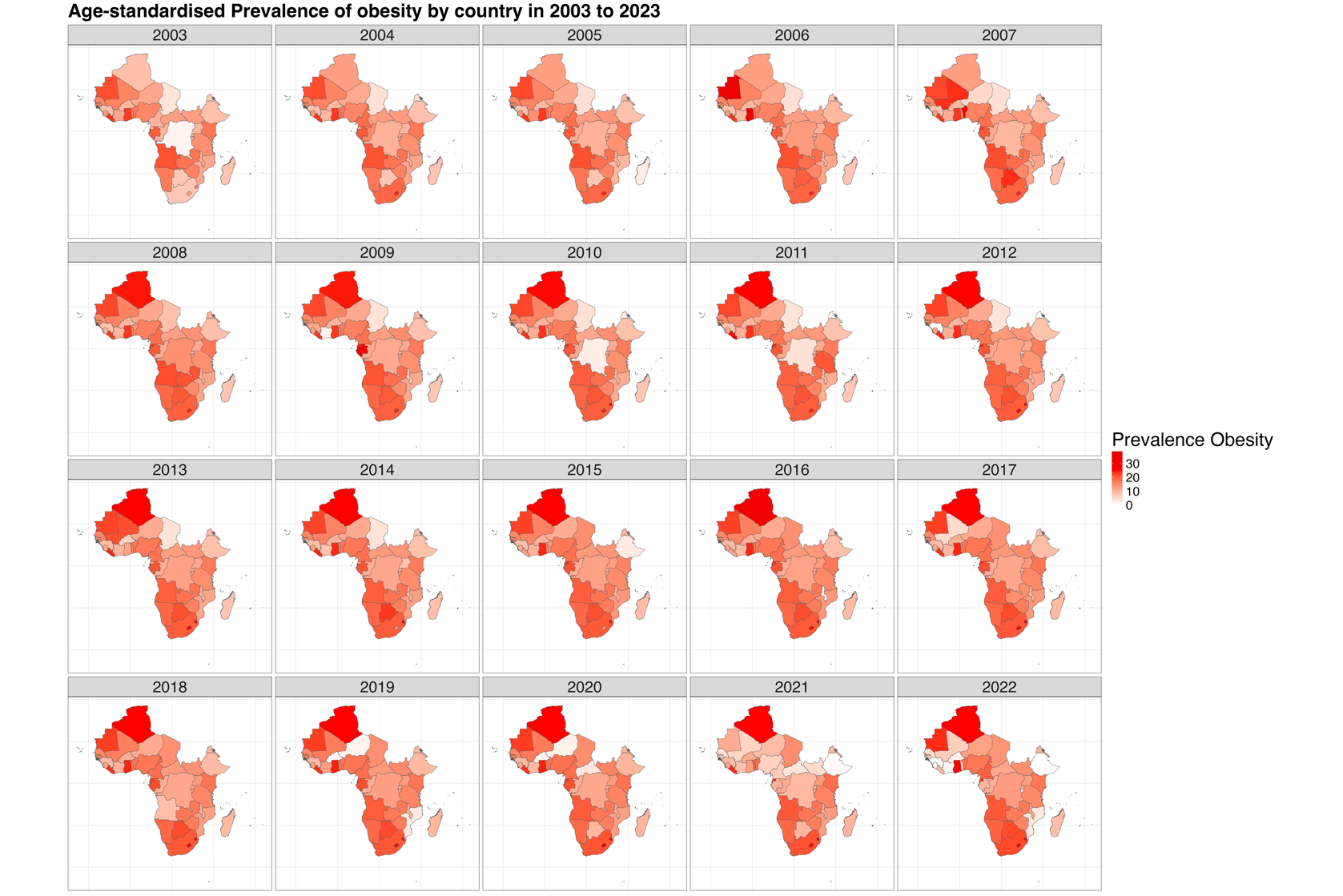


All estimates derive from the spatio-temporal model

## Supplementary Figure 14. Estimated age-standardized prevalence of obesity by country from 2003 to 2022


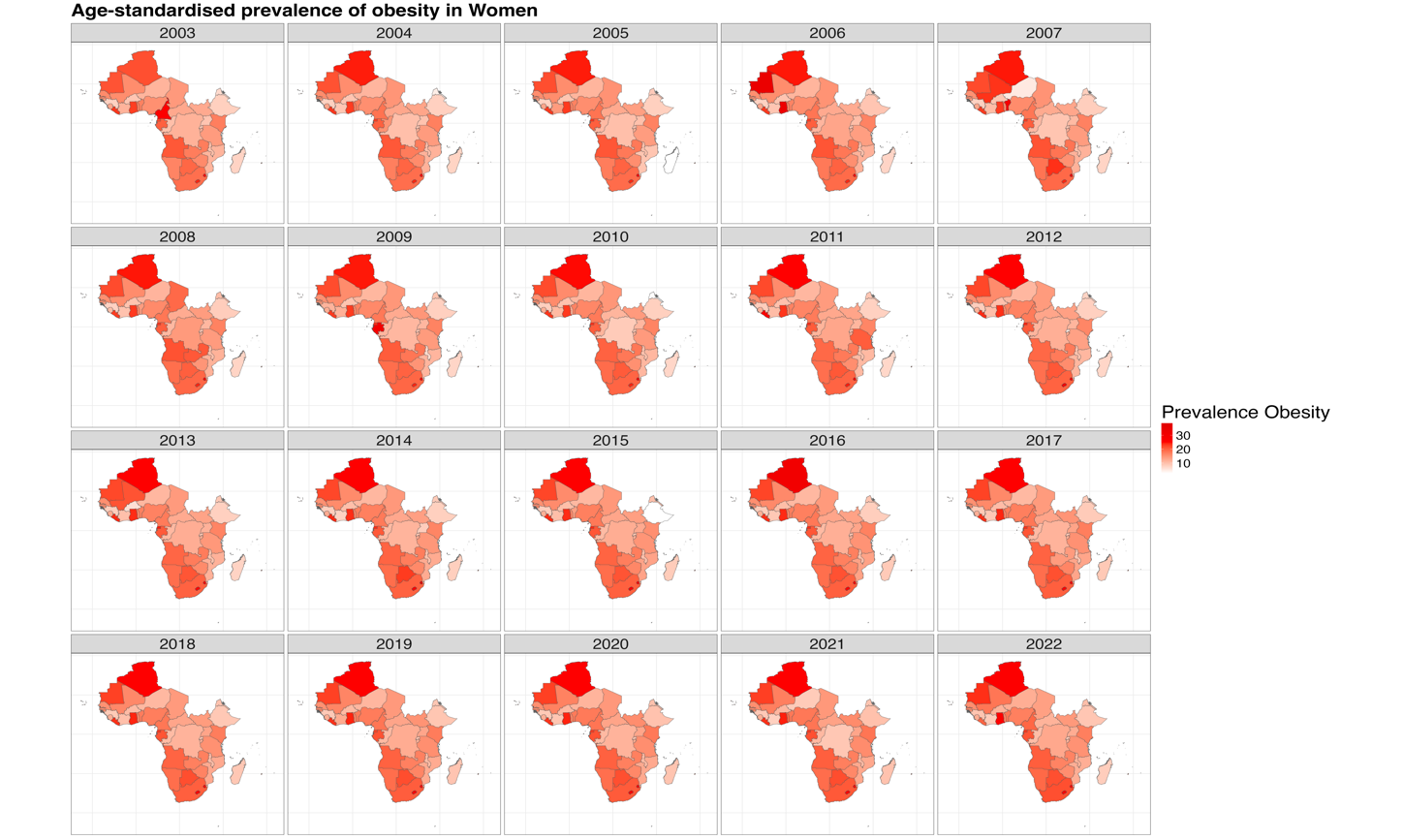


All estimates derive from the spatio-temporal model

## Supplementary Figure 15. Estimated age-standardized prevalence of obesity in women by country from 2003 to 2022


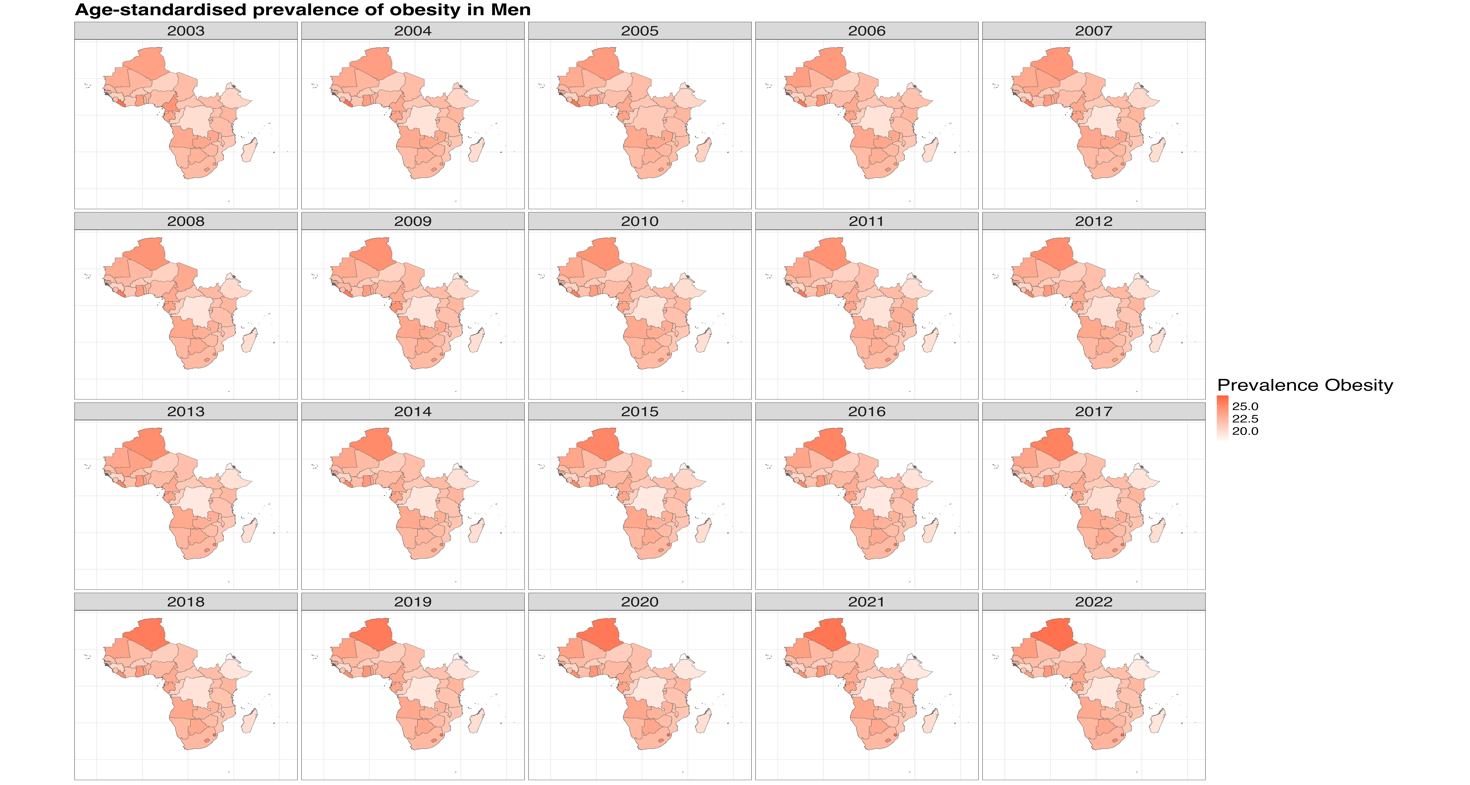


All estimates derive from the spatio-temporal model

## Supplementary Figure 16. Estimated age-standardized prevalence of obesity in men by country from 2003 to 2022


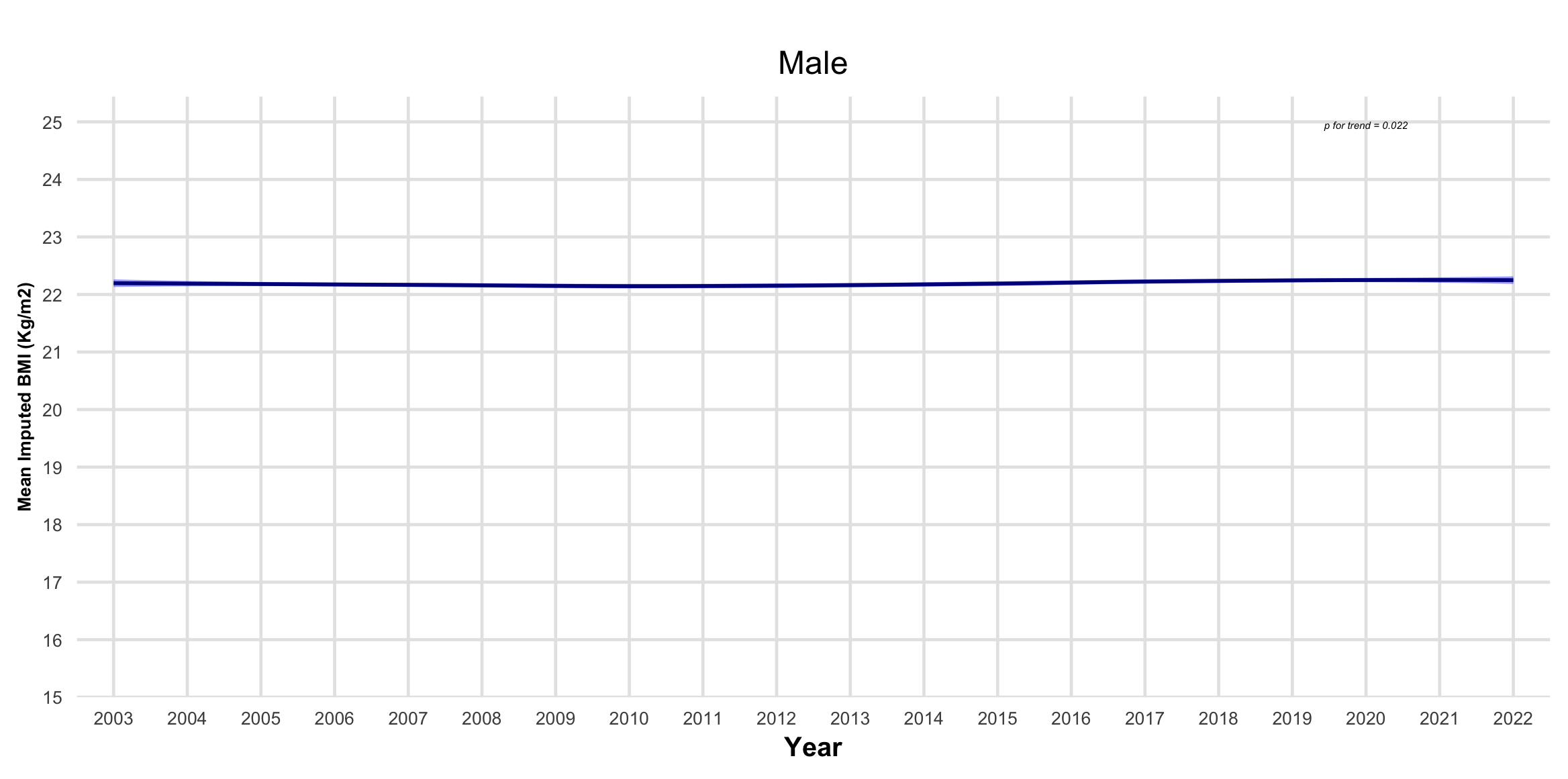


## Supplementary Figure 17. Trend in age-standardised mean BMI from 2003 – 2022 for males. Imputed data for all the 47 countries in the WHO African region


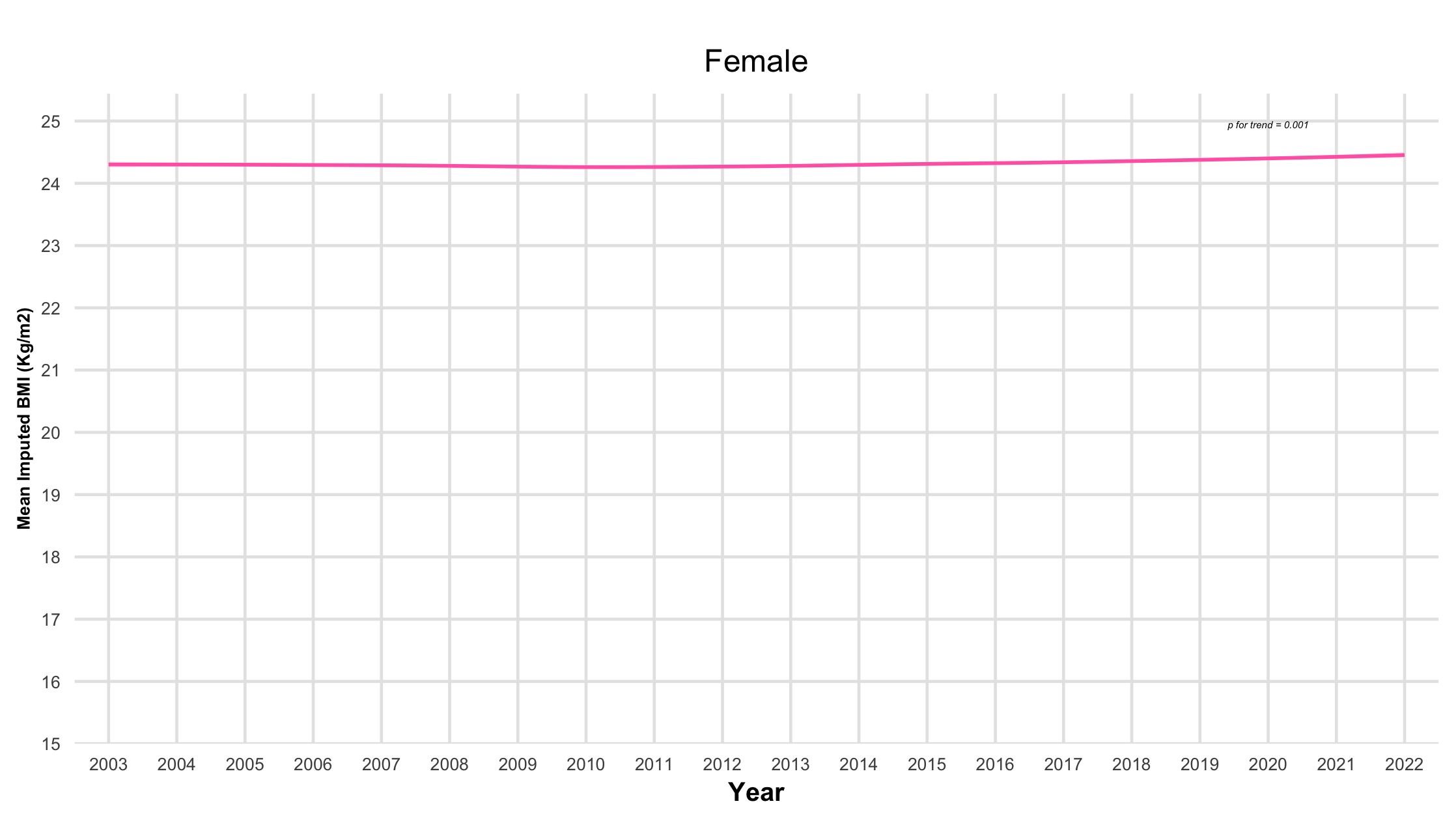


## Supplementary Figure 18. Trend in age-standardised mean BMI from 2003 – 2022 for females. Imputed data for all the 47 countries in the WHO African region

# **References:**

1. WHO. STEPwise approach to NCD risk factor surveillance (STEPS) [Internet]. [cited 2022 Sep 17]. Available from: https://www.who.int/teams/noncommunicable-diseases/surveillance/systems-tools/steps

2. Moraga P. Chapter 7 Spatio-temporal modeling of areal data. Lung cancer in Ohio | Geospatial Health Data: Modeling and Visualization with R-INLA and Shiny [Internet]. [cited 2024 Jun 29]. Available from: https://www.paulamoraga.com/book-geospatial/sec-arealdataexamplest.html

3. Krainski E, Gómez-Rubio V, Bakka H, Lenzi A, Castro-Camilo D, Simpson D, et al. Advanced Spatial Modeling with Stochastic Partial Differential Equations Using R and INLA. New York: Chapman and Hall/CRC; 2018. 298 p.

4. Van Niekerk J, Krainski E, Rustand D, Rue H. A new avenue for Bayesian inference with INLA. Comput Stat Data Anal [Internet]. 2023 May 1 [cited 2024 Jun 28];181:107692. Available from: https://www.sciencedirect.com/science/article/pii/S0167947323000038
